# Cellular and Molecular Processes Are Differently Influenced in Primary Neural Cells by Slight Changes in the Physicochemical Properties of Multicore Magnetic Nanoparticles

**DOI:** 10.1021/acsami.3c02729

**Published:** 2023-03-28

**Authors:** Esther Benayas, Ana Espinosa, M. Teresa Portolés, Virginia Vila-del Sol, M. Puerto Morales, María C. Serrano

**Affiliations:** †, Instituto de Ciencia de Materiales de Madrid, Consejo Superior de Investigaciones Científicas, calle Sor Juana Inés de la Cruz 3, Madrid 28049, Spain; ‡ Departamento de Bioquímica y Biología Molecular, Facultad de Ciencias Químicas, Universidad Complutense de Madrid, Instituto de Investigación Sanitaria del Hospital Clínico San Carlos (IdISSC), Madrid 28040, Spain; § CIBER de Bioingeniería, Biomateriales y Nanomedicina (CIBER-BBN), Instituto de Salud Carlos III (IDSCIII), Madrid 28040, Spain; ∥ Hospital Nacional de Parapléjicos, Servicio de Salud de Castilla-La Mancha (SESCAM), Finca de la Peraleda s/n, Toledo 45071, Spain

**Keywords:** iron oxide nanoparticle, lipidome, magnetic actuation, membrane fluidity, mRNA, primary neural cell

## Abstract

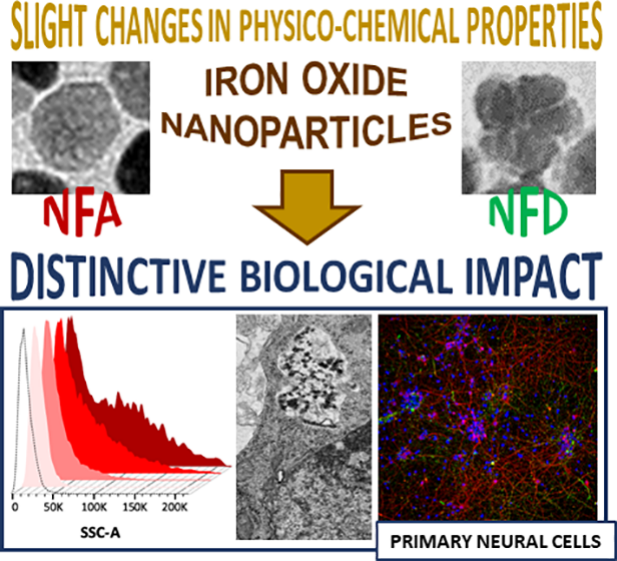

Herein, we use two exemplary superparamagnetic iron oxide multicore nanoparticles (SPIONs) to illustrate the significant influence of slightly different physicochemical properties on the cellular and molecular processes that define SPION interplay with primary neural cells. Particularly, we have designed two different SPION structures, NFA (i.e., a denser multicore structure accompanied by a slightly less negative surface charge and a higher magnetic response) and NFD (i.e., a larger surface area and more negatively charged), and identified specific biological responses dependent on SPION type, concentration, exposure time, and magnetic actuation. Interestingly, NFA SPIONs display a higher cell uptake, likely driven by their less negative surface and smaller protein corona, more significantly impacting cell viability and complexity. The tight contact of both SPIONs with neural cell membranes results in the significant augmentation of phosphatidylcholine, phosphatidylserine, and sphingomyelin and the reduction of free fatty acids and triacylglycerides for both SPIONs. Nonetheless, NFD induces greater effects on lipids, especially under magnetic actuation, likely indicating a preferential membranal location and/or a tighter interaction with membrane lipids than NFA, in agreement with their lower cell uptake. From a functional perspective, these lipid changes correlate with an increase in plasma membrane fluidity, again larger for more negatively charged nanoparticles (NFD). Finally, the mRNA expression of iron-related genes such as *Ireb-2* and *Fth-1* remains unaltered, while *TfR-1* is only detected in SPION-treated cells. Taken together, these results demonstrate the substantial impact that minor physicochemical differences of nanomaterials may exert in the specific targeting of cellular and molecular processes. A denser multicore structure generated by autoclave-based production is accompanied by a slight difference in surface charge and magnetic properties that become decisive for the biological impact of these SPIONs. Their capacity to markedly modify the lipidic cell content makes them attractive as lipid-targetable nanomedicines.

## 1 Introduction

Neural diseases continue to be an enormous challenge for current medicine, as a plethora of physiological and pathological features of the nervous system are still unknowledgeable. The search for novel therapeutics able to assist in the treatment of these pathologies is then mandatory. In this scenario, nanomedicine can provide attractive nanometer-sized solutions with enormous versatility for diagnosis and therapy in the context of modern personalized medicine. Specifically, the use of superparamagnetic iron oxide nanoparticles (SPIONs) in this field has advanced extensively in recent years.^1−3^ Their interesting intrinsic properties postulate them as ideal candidates for a diversity of biomedical applications, such as cell labeling, drug delivery, magnetic hyperthermia, and magnetic resonance imaging, among many others.^2,4^ In the context of neural pathologies, SPIONs are providing attractive opportunities for the development of novel contrast agents for diagnosis imaging, targeted carriers with an enhanced ability to cross the blood-brain and blood-spinal cord barriers for drug delivery, enhancers of magnetic stimulation procedures,^5^ and promoters of neural regeneration,^1^ to cite a few. One of the first attempts in the use of SPIONs for neural regeneration was carried out in the peripheral nervous system.^6^ Since then, magnetite (Fe_3_O_4_) and maghemite (γ-Fe_2_O_3_) nanoparticles have become one of the most commonly used nanomaterials due to their high magnetic susceptibility and biocompatibility, frequently functionalized to either control biodistribution or magnify their physicochemical properties.^4^ An attractive feature of SPIONs for neural therapeutic applications is their capacity to be activated by an external magnetic field.^7−10^ Indeed, neural cells have been found to grow and align in the direction of magnetic gradients and to increase the neurite number and length.^3,7,9^ When applied *in vivo* in an experimental model of Parkinson’s disease in rats,^10^ SPIONs reduced the lesion volume and significantly enhanced mitochondrial function in combination with an electromagnetic field. In a different work, Evans and colleagues genetically engineered primary cortical neurons by using SPIONs without altering cell morphology, viability, and ion channel functioning.^11^

The pivotal role played by parameters such as nanoparticle concentration, size, exposure time, and surface functionalization in SPION interactions with neural cells was first proved by using immortalized cell lines with neuron-like phenotypes. For instance, Marcus et al. examined the response of PC12 cells with different SPIONs (spherical particles with hydrodynamic diameters of 45 ± 17 nm and 99 ± 47 nm before and after functionalization with nerve growth factor, NGF, respectively).^12^ Only uncoated nanoparticles (23 ± 2 nm) were internalized without cytotoxic effects even at high concentrations (up to 0.6 mg Fe mL^–1^). Liu et al. also exposed PC12 cells to uncoated SPIONs (mean diameter of 52 nm),^13^ reporting high viability and neural differentiation at low concentrations (<0.05 mg mL^–1^), but an important reduction in cell survival at higher doses (>0.06 mg mL^–1^). Regarding size, Imam et al. found that SPIONs as small as 10 nm generated neuronal damage in SH-SY5Y cells (i.e., a cell line of neuroblastoma origin) after 24 h of exposure at a very low dose (0.01 mg mL^–1^).^14^ These data already alerted us to the neurotoxic potential of some very small sized SPIONs and the higher susceptibility of neural cells to SPION-mediated toxicity mechanisms in comparison with other cell types. Considering that nanoparticle size is essential to ensure a desired magnetic performance, a compromise must be taken to maximize therapeutic outcomes. Concerning exposure time, cytotoxicity typically increases in a time-dependent manner, even at low nanoparticle concentrations.^15^

SPION functionalization is commonly pursued to benefit cell survival, proliferation, differentiation, internalization, and neurite outgrowth, which is more prompted to occur at low concentrations.^16−19^ Indeed, the specific nature of the coating itself exerts a major influence on neural responses. For example, chondroitin sulfate glycosaminoglycan produced low toxicity and promoted growth of both PC12 and C6 cells (i.e., glial cell strain for glioblastoma research) when tested as SPION coating, even at high concentrations (1 mg mL^–1^).^20^ Functionalization with growth factors such as NGF also increased PC12 cell viability in a dose-dependent manner.^21^ Contrarily, silica and oleic acid coatings of SPIONs led to oxidative stress and decreased viability in SH-SY5Y cells.^22,23^

Moreover, neural cell responses to SPIONs can be optimized by using organic coatings such as poly-l-lysine,^24^ dextran,^25−27^ aminosilane,^27,28^ polydimethylamine,^27^ and dimercaptosuccinate,^29^ among others. Generally, this surface functionalization enables higher rates of internalization and cell survival at low concentrations. However, at higher SPION doses, a significant reduction in cell viability is typically found. This toxicity could derive from the coating material itself, rather than the iron oxide core, as in the case of polydimethylamine.^27^ From the biological point of view, the primary neural cell source, and therefore their specific functional phenotype and culture purity, are also known to largely influence the results obtained.^30^ To minimize these source-related discrepancies, stem cells have been explored. In some of these studies, neural stem cells maintained their proliferation rate and neuronal differentiation capacity when exposed to low concentrations of SPIONs.^31,32^ These nanoparticles even promoted the generation of neural precursors from induced pluripotent stem cells derived from human lung fibroblasts.^33^ When explored *in vivo* in animal models, some formulations of SPIONs, oral or intravenously administered, tended to accumulate in the brain and spinal cord with associated side effects, such as motor discoordination, worsening of clinical signs, local mitochondrial damage, and apoptosis.^34−36^ Work by Su et al. proved the avoidance of such deleterious effects by coating SPIONs with biocompatible molecules such as poly(ethylene glycol), polyethylenimine, and dimyristoylphosphatidylcholine, which even promoted SPION entrance in the myelin sheaths.^37^

To further boost SPION translation into human neural therapeutics, there is a clear need to unravel their specific cell interactions and intracellular effects. In this work, we selected two slightly different multicore SPIONs, with sizes around 20 nm and excellent magnetic properties in terms of magnetic moment per particle and heating capacities,^38^ to better understand the still uncertain role that the physicochemical properties of nanomaterials play in neural cell fate and functioning. Important ingredients to obtain such nanoflowers are the amine and the polyol media, which control the formation of the green rust precursor and provide high viscosity and the possibility of increasing the temperature above 200 °C to form multicore structures, also called mesocrystals.^39^ Two different synthesis routes were compared, one based on an open system and the other based on the utilization of an autoclave, which are expected to help in reproducibility. The effect of the so fabricated SPIONs on diverse cellular and molecular processes in primary neural cells was investigated by a multimethodological approach, including confocal and electron microscopies, flow cytometry, lipidomics, and RT-qPCR. The impact of the combined application of SPIONs with an alternating magnetic field was also explored.

## 2 Materials and Methods

For an extended version of the Material and Methods, please refer to the Supporting Information.

### 2.1 Materials

Reagents for SPION syntheses were purchased from Sigma-Aldrich. Ethanol (96%) was purchased from Scharlau. Other chemical reagents and primary antibodies were purchased from Merck and used as received unless otherwise indicated. Cell culture components, immunofluorescence probes, and secondary antibodies were purchased from Fisher Scientific.

### 2.2 Synthesis and Characterization of Iron Oxide Nanoparticles

The synthesis of multicore SPIONs was based on previous works.^39,40^ Two heating systems were used here, one based on a heating mantle and a glass reactor under reflux and mechanical stirring conditions (NFD), and another based on a Teflon lined stainless steel autoclave (NFA), which is a closed system that allows the minimization of handling. The so prepared nanoparticles were subjected to an acid treatment following a protocol previously reported.^41^ All synthesized magnetic nanoparticles were coated with citric acid. Colloidal properties were analyzed by Dynamic Light Scattering (DLS) in a Zetasizer apparatus (Malvern) to determine the hydrodynamic size (D_hydro_) and surface charge. Core size and morphology were analyzed by transmission electron microscopy (TEM, JEOL JEM 1010). Size distribution was determined by measuring ∼150 particles with ImageJ digital software. Data were fitted to a log-normal curve and the mean value (*D*_TEM_) obtained. Surface and internal compositions were analyzed by energy-dispersive X-ray spectroscopy (EDX) using a field emission scanning electron microscope (SEM, FEI Verios 460) at 2 kV accelerating voltage and a probe current of 13 pA. In addition, Fourier-transform infrared spectroscopy (FTIR) was carried out in a Bruker Vertex 70 V spectrophotometer with 2 cm^–1^ resolutions in KBr pellets. Magnetic characterization was carried out using Vibrating Sample Magnetometer (VSM, Oxford instrument) and SQUID magnetometers (Quantum Design). Hysteresis loops were recorded in the VSM at room temperature after applying a magnetic field of ±5 T to obtain the coercivity (*H*_c_) and the saturation magnetization value (*M*_s_) by extrapolation to infinity field. Zero Field Cooling and Field Cooling (ZFC/FC) magnetization curves were recorded between 300 and 5 K at 100 Oe in the SQUID. The Fe concentration in colloids and cell suspensions was determined by inductively coupled plasma optical emission spectroscopy (ICP-OES) in a PERKIN ELMER OPTIMA 2100 DV apparatus after digestion with nitric acid and aqua regia at 90 °C (1.2 × 10^6^ cells in 10 mL). The heating efficiency was evaluated in a Five Celes apparatus with an Osensa temperature probe, measuring the temperature change under the application of an alternating magnetic field (280 kHz-20 mT and 90 kHz-60 mT) in 1 mL of sample at 20–25 mM Fe concentration. The crystal structure of the sample was identified by X-ray diffraction using a Bruker D8 ADVANCE diffractometer with Cu Kα radiation between 10° and 90° in 2θ. The crystal size was calculated from the (311) peak broadening.

Dynamic light scattering was also used to assess the protein corona formation when NFA and NFD nanoparticles were incubated with fresh (and complete) Neurobasal culture media at different time points (0, 15, 30, 60, and 120 min and 24 h) at 37 °C in a 5 % CO_2_ atmosphere (inside a sterile cell incubator).

### 2.3 Primary Neural Cells Isolation, Culture, and SPION Exposure

Embryonic neural progenitor cells (ENPCs) were obtained from cerebral cortices of Wistar rat embryos, as previously described.^42^ Adult female Wistar rats were provided by the animal facilities of the National Hospital for Paraplegics and sacrificed when gestation reached 16–17 days (E16-E17). All of the experimental protocols for cell collection adhered to the regulations of the European Commission (directives 2010/63/EU and 86/609/EEC) and the Spanish Government (RD53/2013 and ECC/566/2015) for the protection of animals used for scientific purposes. A total of 15 independent cell cultures (*N* ≥ 3 per cell assay and triplicates per culture condition) were carried out, with cell viability being above 85%. Prior to cell culture, plates were coated with poly(l-lysine) (PLL) (45 μg mL^–1^) and later conditioned for 1–2 h in complete culture medium in a sterile incubator at 37 °C under a 5 % CO_2_ atmosphere. Cell seeding density was 25 × 10^3^ cells cm^–2^ for all experiments except for qRT-PCR studies, in which seeding density was doubled to increase the quantity of mRNA per condition. For flow cytometry studies, ENPC suspensions right after isolation were exposed to SPIONs at a density of 100 000 cells mL^–1^ (a total suspension of 5 mL per treatment condition). Cells were maintained for different time points (days-*in vitro*, DIV) in complete Neurobasal culture medium containing B-27 supplement (2 %), streptomycin (100 UI mL^–1^), penicillin (100 UI mL^–1^), and GlutaMAX (1%), which was half-replaced every 3–4 days. At different culture times, as specifically indicated for each type of assay, cells were exposed to either NFA or NFD SPIONs at the different concentrations of selection in a sterile incubator at 37 °C under a 5 % CO_2_ atmosphere. After 24 h of exposure, SPION-exposed cells were gently washed with phosphate buffer saline (PBS) twice and maintained in fresh culture media for another 24 h.

### 2.4 Magnetic Stimulation Studies

For magnetic stimulation, cell culture Petri dishes of 3.5 cm in diameter were used. A total of six different conditions were evaluated: (1) cells without SPIONs nor magnetic field (Control), (2) cells only exposed to an alternating magnetic field (AMF), (3) cells only exposed to NFA SPIONs (NFA), (4) cells only exposed to NFD SPIONs (NFD), (5) cells exposed to both NFA SPIONs and a magnetic field (NFA + AMF), and (6) cells exposed to both NFD SPIONs and a magnetic field (NFD + AMF). ENPC cultures were exposed to the selected concentration(s) of either NFA or NFD SPIONs 48 h before the application of the magnetic field. The hyperthermia treatment of selection was an alternating magnetic field of 20 mT for 60 min, with a frequency of 280 kHz, applied by using Five Celes equipment.

### 2.5 Morphological Studies by Electron Microscopies

Cell culture morphology after exposure to SPIONs was first studied by using a field-emission Philips XL30 S-FEG scanning electron microscope (FESEM). SPION internalization in ENPCs was characterized both in culture and in suspension by transmission electron microscopy (TEM). For the visualization of SPIONs internalized by cells in culture, ENPCs were seeded on Permanox chambers (Nunc Lab-Tek) for 14 DIV. At 12 DIV, cells were treated with either NFA or NFD SPIONs at different concentrations. Samples were then prepared for TEM and visualized by using a Jeol JEM 1010 microscope (Japan) at 80 kV with a coupled camera (Gatan SC200, USA). For the visualization of SPION uptake by cells in suspension, ENPCs right after isolation were exposed to either NFA or NFD at different concentrations for 4 h at 37 °C. Cell suspensions were then centrifuged at 300 g for 4 min, and the obtained cell pellets were prepared for TEM and examined at 100 kV in a Jeol JEM 1400 Flash (Tokyo, Japan) microscope. Pictures were taken with a OneView (Gatan) digital CMOS camera (4K × 4K). Images were acquired in both bright and dark field modes to clearly identify SPIONs inside the cells.

### 2.6 Viability Studies by Confocal Fluorescence Microscopy

Cell viability in culture was analyzed using a Live/Dead viability kit according to the manufacturer’s instructions (Life Technologies). After staining, the samples were visualized by using a Leica SP5 confocal laser scanning microscope. Collected images (*N* ≥ 5 per condition) were analyzed using the ImageJ software to quantify the area occupied by those positively stained for each marker with respect to the total image area.

### 2.7 Viability and Internalization Studies by Flow Cytometry

First, flow cytometry studies were carried out to analyze cell viability and the effect of SPION internalization on cell size and complexity in ENPC suspensions. Conditions investigated included: (1) cells without SPIONs (control), (2) cells exposed to NFA SPIONs at 4 different concentrations (0.001, 0.01, 0.025, and 0.05 mg Fe mL^–1^), and (3) cells exposed to NFD SPIONs at 4 different concentrations (0.001, 0.01, 0.025, and 0.05 mg Fe mL^–1^). Exposure times were 1, 2, 4, and 24 h in a sterile incubator at 37 °C under a CO_2_ atmosphere (5 %). By using a commercial kit containing Annexin V and 7AAD probes (Beckman Coulter Life Sciences), we were able to differently labeled subsets of live (Annexin^–^/7AAD^–^), early apoptotic (Annexin^+^/7AAD^–^), late apoptotic (Annexin^+^/7AAD^+^) and dead (Annexin^–^/7AAD^+^) cells (Figure S1). Samples were analyzed on a FACS Canto II cytometer (BD Biosciences) within 30 min after staining and recorded for 2 min with at least 10 000 events recorded in the FSC gate. Flow cytometry data analysis was carried out with the FlowJo 10.7 software (BD Biosciences).

For SPION uptake studies, ENPC suspensions were preincubated for 2 h in a sterile incubator at 37 °C under a CO_2_ atmosphere (5 %) with the following inhibitors: (i) chlorpromazine, (ii) amiloride, (iii) cytochalasin D, (iv) genistein, and (v) wortmannin. After inhibitors preincubation, cell suspensions were then exposed to either NFA or NFD SPIONs for 2 h. After corresponding incubations, cell suspensions were prepared for flow cytometry analyses as described above. Cell viability and neural differentiation was analyzed after 7 DIV by confocal fluorescence microscopy as described in other sections.

### 2.8 Neural Differentiation by Confocal Fluorescence Microscopy

An immune-labeling procedure was used to investigate the impact of SPIONs exposure on neural cell differentiation by using primary antibodies against MAP-2 and β-III tubulin for labeling neurons and vimentin for labeling non-neuronal cells including glia and glial fibrillary acidic protein (GFAP) for specific targeting of astrocytes. Additionally, synaptophysin, the most abundant protein in the membrane of synaptic vesicles, was used to visualize synapses in neurons. Appropriate secondary antibodies were selected. Cell nuclei were stained with Hoechst. Samples were visualized using a Leica TCS SP5 microscope. Capture conditions in each case were established by using appropriate positive and negative controls and maintained during the acquisition of all the images. Collected images (*N* ≥ 5 per condition) were analyzed using ImageJ software. Both the area and the number of cells positively stained for each marker were quantified and normalized by cell density (obtained from Hoechst images).

### 2.9 Lipidome Studies

ENPC cultures on Petri dishes (3.5 cm in diameter) were exposed to different conditions and then trypsinized. EquiSPLASH was used as an internal standard. Diluted extracts (500 μL) were analyzed through direct infusion in an ESI qQTOF (TripleTOF 6600+, Sciex) mass spectrometer equipped with a DuoSprayTM source (Sciex). Samples were analyzed in both positive and negative ions mode with the MSMSALL acquisition mode, consisting of a TOF Ms scanning mode and a series of MSMS scanning modes stepped in mass intervals of 200–1200 umas. Data analysis was carried out by using LipidView v1.3 software. Lipid classes and species were identified based on the basis of *m*/*z* exacta and fragmentation patterns. Further details on the lipidome studies performed are provided in the Supporting Information.

### 2.10 Membrane Permeability Studies by Flow Cytometry

The effect of SPIONs on cell membrane permeability was analyzed using two fluorescent dyes: FM 1-43 and Laurdan. Conditions investigated included: (1) cells without SPIONs and without dyes (control), (2) cells exposed to NFA SPIONs without dye, (3) cells exposed to NFD SPIONs without dye, (4) cells loaded with FM1-43, (5) cells exposed to NFA SPIONs and loaded with FM1-43, (6) cells exposed to NFD SPIONs and loaded with FM1-43, (7) cells loaded with Laurdan probe, (8) cells exposed to NFA SPIONs and loaded with Laurdan, and (9) cells exposed to NFD SPIONs and loaded with Laurdan. Briefly, right after isolation, ENPC suspensions were exposed to NFA and NFD SPIONs (0.05 mg mL^–1^) for 2 h in a sterile incubator at 37 °C under a CO_2_ atmosphere (5 %). After treatment, cells were centrifuged at 300 *g* for 4 min, the supernatants were removed, and the pellets were suspended. For FM1-43 staining, cells were filtered and centrifuged at 300 *g* for 4 min at 4 °C. Next, supernatants were discarded, and pellets were suspended in 1 mL of Hank’s without sodium and magnesium. Subsequently, 2.5 μL of FM1-43 (2.5 μg mL^–1^) was added to the samples and incubated for 1 min. Labeled samples were then analyzed by using flow cytometry. FM1-43 fluorescence was excited with a 488 nm laser and the emission fluorescence was collected with a B585/42 detector in the 564–606 nm. For Laurdan staining, cell pellets were incubated with 5 μL of Laurdan (5 μM) for 1 h at 37 °C. Then, cells were filtered and centrifuged at 300 *g* for 4 min at 4 °C. Supernatants were discarded, pellets were suspended in 1 mL of DMEM and measured by flow cytometry immediately after. Laurdan fluorescence was excited with a 405 nm laser and the fluorescence emission was detected with two different detectors: V450/50 for collecting emission at 425–475 nm and V530/30 for collecting emission at 515–545 nm. The ratio between the emissions at 530/30 and 450/50 was calculated. General Polarization (GP) values were calculated using the median of fluorescence intensity (MFI) emission values at 450 and 530 nm and excitation at 405 nm, according to the formula ^405^GP_ex_ = (MFI_450_ – MFI_530_)/(MFI_450_ + MFI_530_). All samples were analyzed by using a FACS Canto II cytometer (BD Biosciences) within 30 min after staining and recorded for 2 min with at least 10 000 events recorded in the FSC gate. Flow cytometry data analysis was carried out by using the FlowJo 10.7 software (BD Biosciences). Further details on the gating strategy followed are provided in Figures S1, S2, and S3.

### 2.11 RT-qPCR Studies

RNA concentration and purity were quantified in a Nanodrop One spectrophotometer (Thermo Fisher Scientific) and a Quantus fluorometer (Promega Corporation). Yield range was between 40.35–75.35 ng μL^–1^ and 39.20–82.20 ng μL^–1^, respectively. Retrotranscription (RT) reactions were performed using the iScript cDNA Synthesis kit (Biorad PN170-8891) following manufactureŕs instructions. The genes of interest (GOIs) analyzed were *Fth1*, *Ireb2*, *Slc11a2*, and *Tfrc*. The putative reference gene analyzed was 18S. The ValidPrime Universal kit was used as a control for genomic background. Specific details of the RT-qPCR studies performed are provided in the Supporting Information and primer sequences used are included in Table S1. Data processing was carried out using the software GenEx v. 5.4.4 (MultiD Analyses AB, Gothenburg, Sweden), performing the following steps: (1) efficiency correction; (2) average technical qPCR replicates, (3) normalization with selected reference gene, and (4) relative quantification 2^–Δ(Δ*C*_q_)^,^43^ where Δ*C*_q_ is the *C*_q_ value of each individual sample against the *C*_q_ value of calibrator group (the first biological replicate of the NFA SPION group). An appropriate normalization strategy is essential to correct the experimental variability (e.g., integrity differences and pipetting errors). In this study, the putative reference gene analyzed was 18S.

### 2.12 Statistics

Results were expressed in conventional bar graphs as the mean ± standard error of the mean (SEM), unless otherwise indicated, of at least three independent experiments for each assay (*N* ≥ 3). Statistical analysis was performed by using IBM SPSS Statistics software (ver. 28.0.1.0). Comparisons among groups were done by one-way analysis of variance (ANOVA) followed by either posthoc Scheffé, Tukey HSD or Games-Howell tests (homogeneous vs heterogeneous variances as dictated by Levene’s test). Comparisons between two groups, when needed, were carried out by *T* test. In all cases, the significance level was defined as *p* < 0.05.

## 3 Results and Discussion

**Figure 1:**
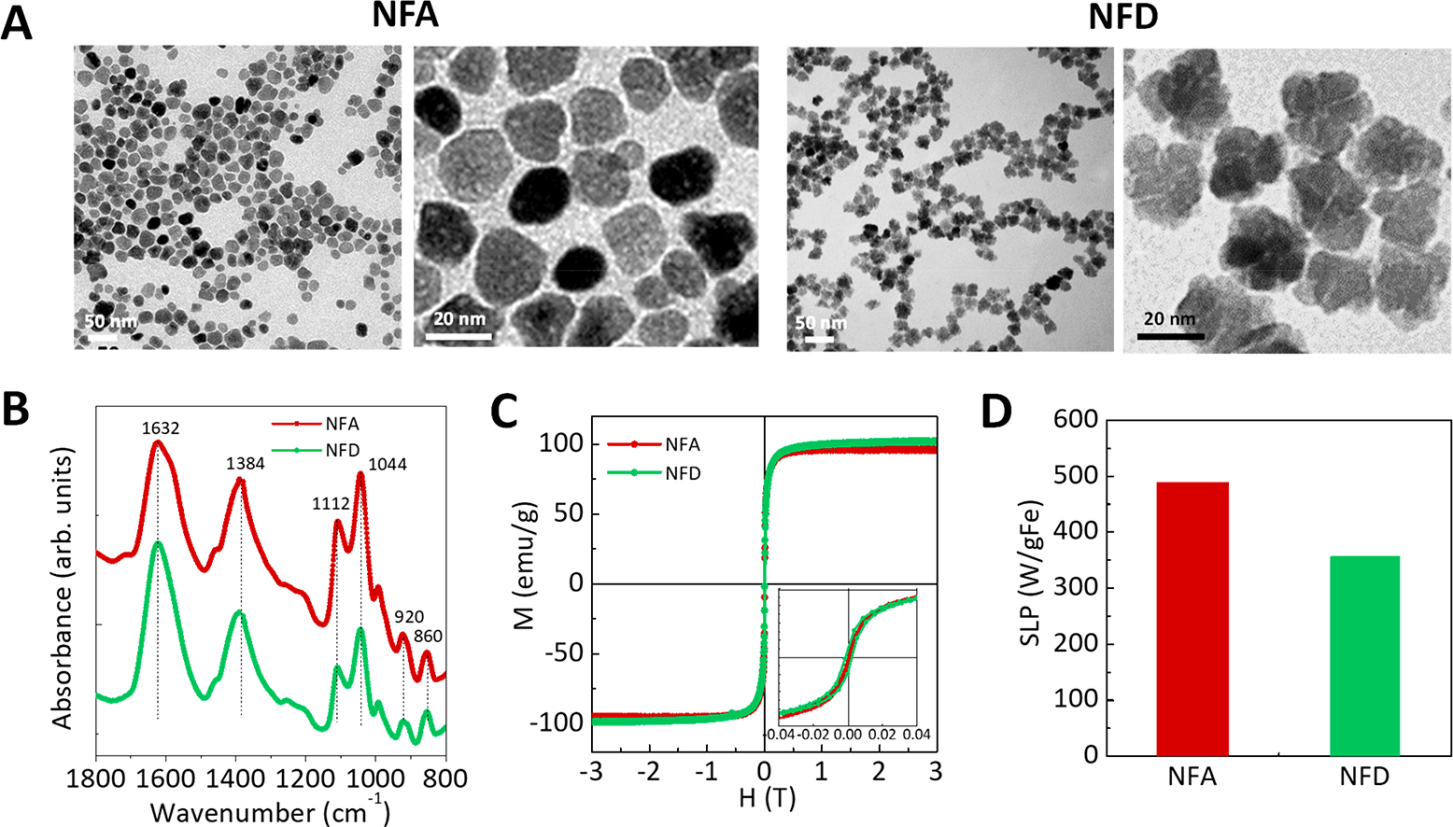
Characterization of the multicore SPIONs prepared in a closed (autoclave) or open system: NFA (red) and NFD (green), respectively. (A) Representative TEM micrographs. Scale bars: 50 nm (left) and 20 nm (right). (B) Infrared spectra in the range 1800 cm^–1^ to 800 cm^–1^ normalized to the Fe–O bands. (C) Hysteresis loops obtained at 300 K and (D) heating efficiency (SLP, W/g_Fe_) under an alternating magnetic field (280 kHz, 20 mT).

By using either an autoclave-based closed or an open system, two different multicore SPIONs were fabricated: NFA and NFD, respectively (Figure 1 and Table 1). These heating routes and the final acid treatment led to maghemite (γ-Fe_2_O_3_) nanoparticles (Figure S4A) with different degree of fusion between cores (crystal size 20 nm for NFA and 15 nm for NFD), but with similar final particle size as obtained by TEM (20.5 ± 6.0 nm for NFA and 20.8 ± 2.0 nm for NFD) (Figure 1A). The growth mechanism proposed for these multicore particles explains how mesocrystals composed of crystallographically oriented primary particles evolve into single large nanocrystals at longer heating times or when heated in a more efficient way, as is the case for the autoclave. After citric acid coating, hydrodynamic sizes increased to around 60 nm for both samples as measured by DLS (63 ± 28 nm and 59 ± 27 nm, for NFA and NFD, respectively, Figure S4B), but NFA presented a slightly less negative surface charge than NFD (−22 mV vs −31 mV), suggesting a lower density of carboxyl groups. We hypothesize that such slight changes in hydrodynamic size and zeta potential may result in important alterations in cell media stability, cytotoxicity, and cellular uptake. Our group has previously reported that large and significant changes in the physicochemical properties of SPIONs dramatically influence intracellular trafficking and degradation in endolysosomes, these responses being dependent on cell type.^44^ EDX and FTIR spectra of the synthesized SPIONs endorsed the only presence of citric acid, and some polyol rests at the nanoparticle surface (Figure 1B and Figure S5). When normalized to the Fe–O bands, the peaks assigned to the citric acid (1632 and 1384 cm^–1^) had a higher intensity for NFD than NFA due to the presence of a larger amount of coating, which is responsible for their larger particle surface charge.

**Table 1:** Main Physicochemical Properties of the Two Different Multicore SPIONs Investigated: NFA and NFD^a^

	*D*_TEM_ (nm)	*D*_XRD_ (nm)	*D*_hydro_ (nm)	Surface charge (mV)	*M*_s_ (emu/g_Fe_) at 300 K	*H*_c_ (Oe) at 300 K	SLP (W/g_Fe_)
**NFA**	20.5 ± 6.0	20 ± 1	63 ± 28	–22	96	10	487
**NFD**	20.8 ± 2.0	15 ± 1	59 ± 27	–31	109	20	347

a SLP was measured at 280 kHz and 20 mT.

Both nanostructures exhibited high saturation magnetization *M*_S_ values (96 emu/g_Fe_ for NFA and 109 emu/g_Fe_ for NFD) and low *H*_C_ (<25 Oe) at 300 K, characteristic of superparamagnetic spinel structures (Figure 1C). However, a different behavior was observed in the ZFC/FC curves, with NFA having the highest blocking temperature, above 300 K (Figure S4C). This finding supports the fact that NFA SPIONs presented a denser structure due to autoclave-based heating. The heating efficiency (specific absorption rate, SLP) of NFA and NFD suspensions was obtained at 280 kHz and 20 mT and at 90 kHz and 60 mT (Figure 1D and Figure S4C), demonstrating superior heating capabilities for these multicore particles (SLP > 300 W/g_Fe_) than for single core ones (SLP < 100 W/g_Fe_).^39^ Again, NFA SPIONs displayed higher values due to their denser structure in comparison to NFD, therefore proving a more effective exchange coupling between cores. Taken together, the use of autoclave heating allowed for the synthesis of SPIONs with a denser multicore structure responsible for a smaller surface area containing fewer citrate groups (i.e., less negatively charged) and higher magnetic responses (i.e., NFA SPIONs). In turn, NFD SPIONs showed a less compact multicore structure that supported a larger surface area allowing a larger number of citrate groups at the surface (i.e., more negatively charged) and lower magnetic responses. It is important to note that the different degree of fusion among cores in the resulting SPIONs was inevitably accompanied by changes in the surface area and therefore in the amount of carboxyl groups decorating it as well as in the magnetic properties.

**Figure 2:**
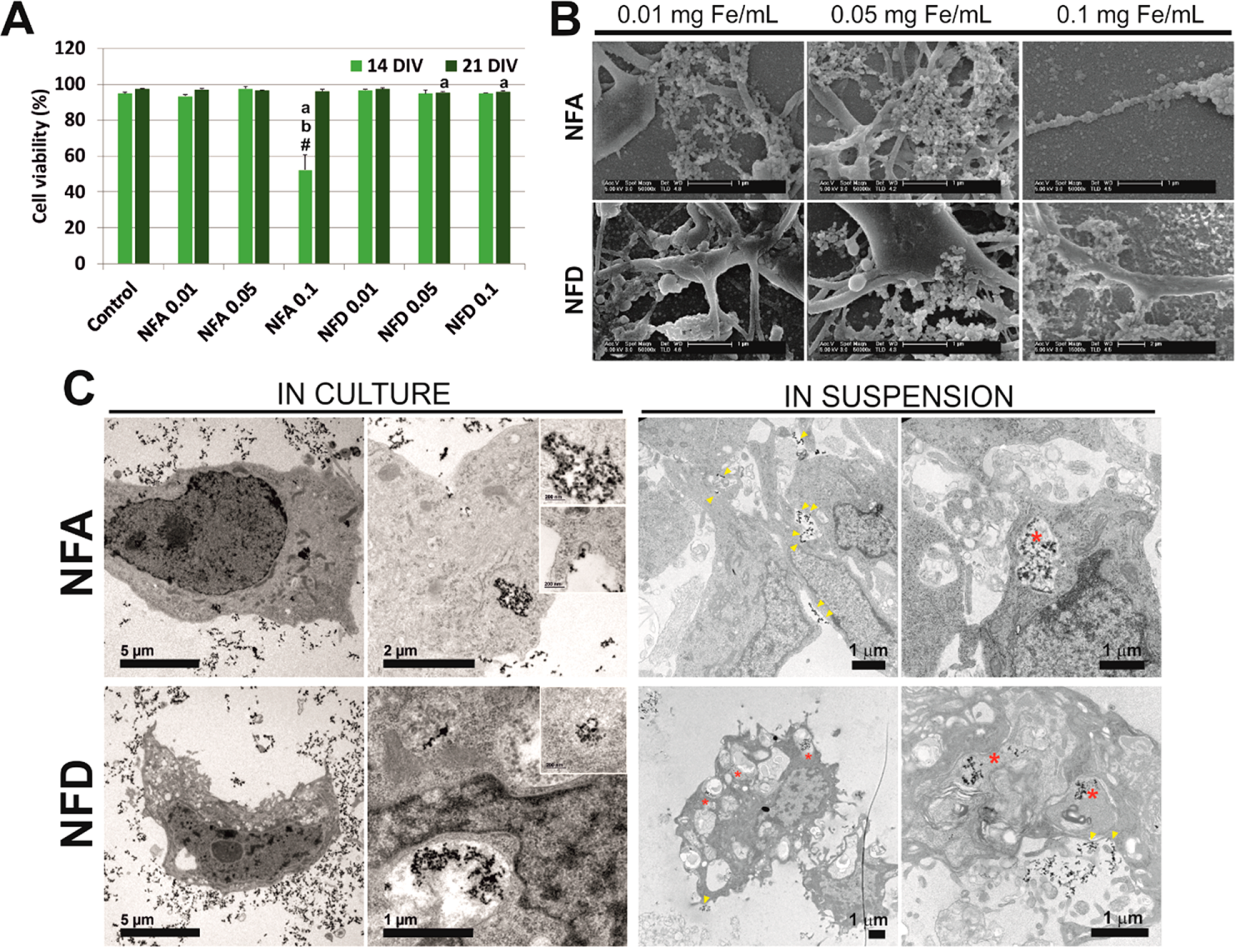
Biocompatibility assessment of NFA and NFD SPIONs at different concentrations in primary neural cells. (A) Viability values expressed as a percentage of the total area labeled by cells. Statistics: one-way ANOVA followed by either *Scheffé* as dictated by the *Levene’s* test. Significant *p* values (*p* < 0.05) with respect to control (*a*), 0.01 and 0.05 mg Fe mL^–1^ of the same SPION type (*b*) and 21 DIV (#). (B) Representative SEM images of ENPC cultures exposed to SPIONs. (C) Representative TEM images of ENPCs exposed to SPIONs in culture (14 DIV) and suspension (24 h). The locations of NFA and NFD at the cell membrane are labeled with yellow head arrows and in intracellular compartments with red asterisks. Insets illustrate intracellularly located SPIONs and receptor-mediated internalization processes. Scale bars are indicated in each image.

These two types of multicore SPIONs were next interfaced with culture media and primary neural cells to study their influence on cellular and molecular processes and the biological impact of their small differences in physicochemical properties in these highly sensitive cell types. First, we analyzed the evolution of the hydrodynamic size when NFA and NFD nanoparticles were incubated with fresh (and complete) Neurobasal culture media at different times, observing an increase of up to around 200 nm for NFA and up to 500 nm for NFD (Figure S6), probably due to their higher surface charge. Differences in the hydrodynamic size of nanoparticles likely relate to changes in their protein corona and then affect endocytic pathways.^45^ It should be mentioned that controlling the protein corona formation is highly complex, since it is a dynamic process that depends on many different factors. Further studies are needed to fully elucidate the composition and evolution of the protein corona of these SPIONs when in contact with the biological milieu. To define safe conditions of SPION exposure avoiding toxic effects,^4,14,16,19,46^ cell viability was next assessed at both 14 and 21 DIV (Figure 2A and Figure S7). Viability in all culture conditions was found statistically similar to control cells without nanoparticles, except for the highest concentration of NFA (0.1 mg Fe mL^–1^), in which a significant reduction was identified (one-way ANOVA followed by Scheffé test, *p* < 0.001*** for all comparisons). Interestingly, this toxic effect reverted at 21 DIV (*p* = 0.376 with respect to control and *p* = 0.727 with respect to the similar concentration of NFD). This finding might be pointing out toward a neuron-dependent toxicity as ENPC cultures are composed of a majority of neurons at 14 DIV, but glial cells become more abundant at 21 DIV under these culture conditions. Statistically significant differences were also found at 21 DIV in NFD-exposed cells at both 0.05 mg of Fe mL^–1^ (*p* = 0.008**) and 0.1 mg of Fe mL^–1^ (*p* = 0.04*) with respect to control cells. This finding may be indicative of the slow initiation of some long-term toxicity mechanisms induced by this type of SPIONs. Morphological details of the SPION-cell interaction in culture were next investigated by SEM (Figure 2B and Figure S8). Both types of SPIONs were found in close contact with the extracellular matrix and neural cell membranes, often forming small aggregates. Further analyses by TEM corroborated both their tight interaction with neural cell membranes and their internalization in culture and in suspension (Figure 2C). Specific internalization routes could not be assessed by this method, but high-magnification images evidenced both membrane-dependent and nondependent intracellular locations (insets in Figure 2C). The use of dark field conditions allowed us to clearly identify SPIONs as brighter elements due to their crystallinity (Figure S9A). For these studies, SPION suspensions and ENPCs were used as reference (Figure S9B).

We next used flow cytometry to deepen on SPION-induced effects on cell viability, discarding the highest concentration tested (0.1 mg of Fe mL^–1^) due to the identified toxicity. The first observation was a significant increase in cell debris (commonly associated with necrosis) at 24 h in those suspensions exposed to concentrations higher than 0.025 mg Fe mL^–1^ of NFA and 0.05 mg Fe mL^–1^ of NFD (concentration dependent) but not at shorter incubation times (Figure S10). After debris being discarded, concentration- and time-dependent toxicity effects were again observed for both SPIONs (Figure S11). Specifically, cell viability decreased with respect to control cells at the highest SPION concentrations (>0.025 mg Fe mL^–1^) and longest exposure times (i.e., 24 h). Again, cells exposed to NFA SPIONs (e.g., slightly less negatively charged) were more largely affected than those with NFD. Early apoptosis was markedly increased with respect to control cells in all concentrations and incubation times for both SPIONs, except for 24 h, at which time it significantly diminished. Late apoptosis only increased over the control value at the shortest time of incubation (i.e., 1 h), probably due to an exacerbation of the cell membrane damage derived from the enzymatic isolation process caused by the SPIONs. Finally, cell death was only significantly augmented after 24 h of SPION exposure, in accordance with the larger abundance of cell debris found. This trend was more dramatic and dependent on concentration for NFA than for NFD. Nonetheless, it is worth noting that absolute values of dead cells were remarkably low (≤0.31% of total cell population) for all SPION conditions. Taken together, these data revealed detrimental effects on neural cell viability dependent on both concentration and exposure time (i.e., the highest the SPION concentration and exposure time, the lowest the viability and early apoptosis percentages), with a differential impact linked to the type of SPION, being larger for NFA than NFD. This deleterious impact on neural cell viability agrees with previous reports for other types of SPIONs,^25,26^ with the highest mortality rates always associated with the most extreme conditions tested. The slightly less negative surface charge of NFA with respect to NFD is hypothesized to drive this differential impact by mediating a larger cell internalization as corroborated by ICP-OES measurements. Specifically, cell uptake of NFA increased from 12 to 19 pg Fe/cell with the incubation time (0.01 mg Fe mL^–1^; 2 and 24 h, respectively) and up to 52 pg Fe/cell at larger concentrations (0.05 mg Fe mL^–1^). For NFD, cell uptake was significantly lower, increasing from 5 up to 10 pg Fe/cell with the incubation time and up to 21 pg Fe/cell at larger concentrations.

**Figure 3:**
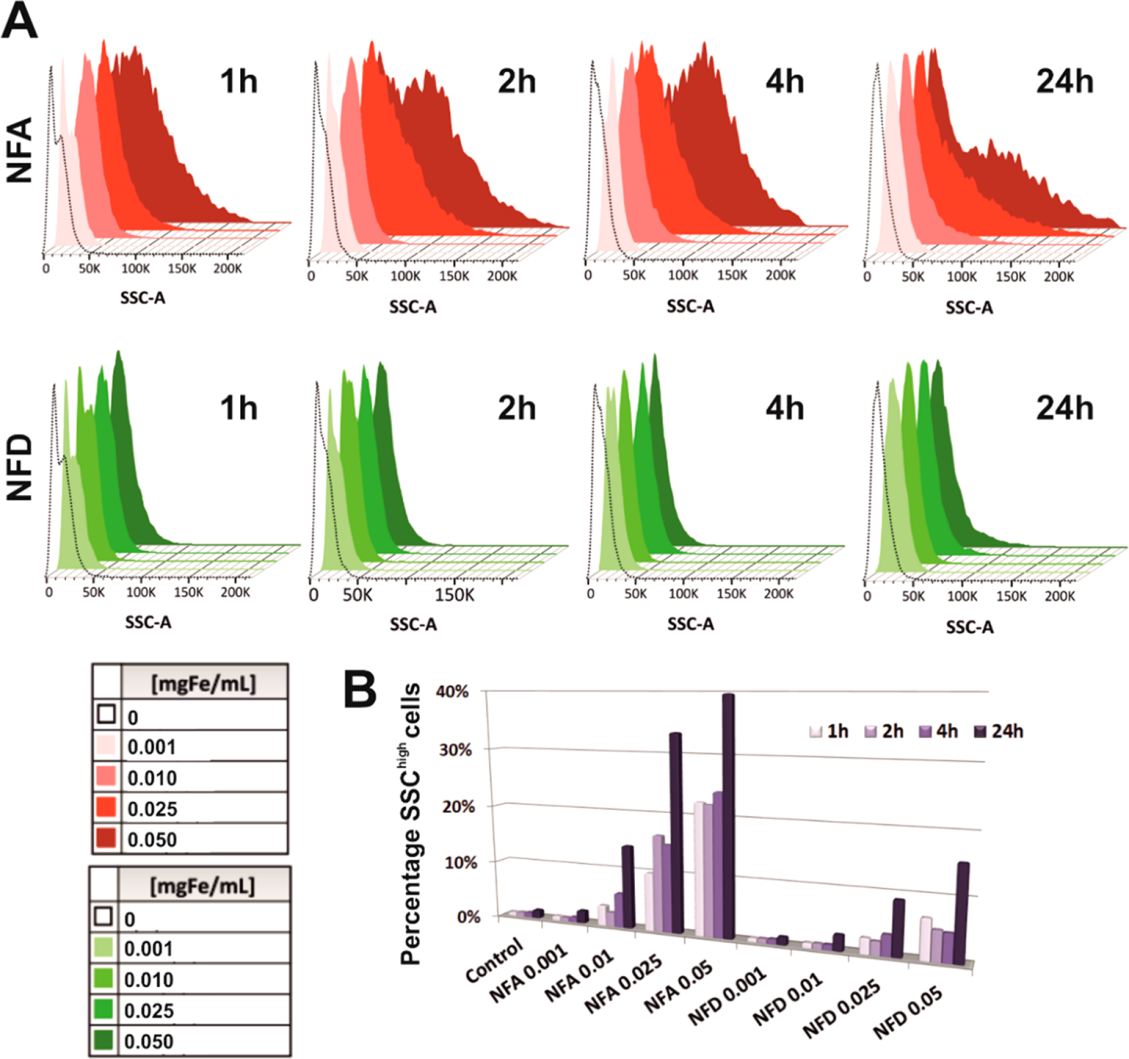
Characterization of the combined effect of SPION type, concentration, and exposure time in primary neural cell complexity by flow cytometry. (A) Histogram overlays of SSC values in area (SSC-A). (B) Percentage of cells in the SSC^high^ subset.

Early in these flow cytometry studies, we noticed a clear alteration of both cell size (measured by the forward scatter, FSC) and, more pronouncedly, cell complexity (measured by the side scatter, SSC) on ENPC suspensions exposed to these SPIONs (Figure S12A). These changes were markedly dependent on nanoparticle concentration, evident at different cell concentrations (i.e., 5 × 10^5^ cells mL^–1^ and 10^6^ cells mL^–1^) and more profound for NFA than for NFD SPIONs. Experiments with murine L929 fibroblasts (highly proliferative, moving, and polygonal shaped cells in contrast to neuron cells) confirmed similar trends for all parameters tested (Figure S12B). When investigated more in detail, the first evidence was the segregation of the initial cell population (FSC gated) in two distinct cell subsets: (1) Cells with normal FSC values and (2) cells with reduced FSC values (FSC^low^) (Figure S1). Specifically, longer incubation times and higher SPION concentrations led to a larger subset of FSC^low^ cells, with a clear superiority for NFA. Within these FSC^low^ cells, complexity gradually increased on the basis of SPION incubation conditions (Figure 3A), thus defining a novel SSC^high^ cell subpopulation following the same trends. When quantified (Figure 3B), these effects were clearly noted. Particularly, ENPC suspensions exposed to 0.05 mg of Fe mL^–1^ of NFA for 24 h showed the larger SSC^high^ subpopulation (40%). We hypothesized these SSC^high^ cells being more closely interacting with SPIONs by a closer contact at the cell membrane and/or larger internalization. Similar SPION type, concentration, and time dependent trends were corroborated for cell size, although the magnitude of these effects was limited in comparison to cell complexity (Figure S13).

We next investigated the capacity of ENPC suspensions to form neural networks after exposure to SPIONs. SPION-treated cell suspensions (0.05 mg Fe mL^–1^ for 2 h) were cultured on PLL-coated Petri dishes and maintained for 7 DIV. Despite the early and late apoptosis previously identified in the suspensions, SPION-treated ENPCs were able to properly adhere and grow neurites in culture (Figure S14A–C). Cell viability was preserved in comparison to control cells. Indeed, NFA-treated cells increased the area of live cells with respect to NFD-treated cultures (*p* = 0.042*) but not with the control (*p* = 0.074). The number of live cells remained unaltered (*p* = 0.338), thus denoting an enhancement of the spreading capability of the NFA-treated cells. Moreover, the area of dead cells in SPION-treated cultures was reduced (*p* = 0.004*** for NFA and *p* = 0.005** for NFD in comparison to control samples). Regarding neural cell differentiation (Figure S14D–J), both SPION-treated neural cell suspensions differentiated into neurons in culture (positive for MAP-2), with similar areas (*p* = 0.692; Figure S14E), cell numbers (*p* = 0.832; Figure S14G) and quantity of neurite intersections (*p* = 0.805; Figure S14I). For non-neuronal cells including glial cells (positive for vimentin), the area occupied by these cells diminished in SPION-treated cells with respect to the control (*p* < 0.001*** for NFA and *p* = 0.005*** for NFD; Figure S14F). This effect was accompanied by a nonstatistically significant reduction of both cell number (*p* = 0.348; Figure S14H) and amount of protrusion intersections (*p* = 0.08; Figure S14J). This favorable neural culture development, despite the presence of Annexin V^+^ cells in the original suspensions, points toward a reversion of early apoptosis stages after removal of the apoptotic stimuli that represent being in suspension for naturally adherent cells.^47,48^ Moreover, both SPIONs seem to display a selective affectation of non-neuronal cell development, as proven by their more dramatic impact on vimentin^+^ cells (or their corresponding progenitors) rather than MAP-2^+^ cells.

Our next step was to elucidate if NFA and NFD SPIONs were entering ENPCs and, if so, by which internalization route(s). Magnetic nanoparticles are typically internalized by active processes such as endocytosis,^7,8,49^ as they are generally too large to easily diffuse across cell membranes. Indeed, different endocytic pathways can be involved depending on their specific physicochemical characteristics.^50^ In this work, we selected five different inhibitors: amiloride, chlorpromazine, cytochalasin D, genistein, and wortmannin, being each of which is able to alter one of the main endocytic mechanisms. Amiloride is thought to indirectly inhibit the process of micropinocytosis by blocking the Na^+^/H^+^ exchanger pump of the plasma membrane, which alters the formation of the macropinosomes.^51,52^ Chlorpromazine blocks the function of the AP2 adaptor, which is one of the key proteins involved in the formation of clathrin-coated pits in clathrin-dependent endocytosis.^51,53^ Cytochalasin D also inhibits micropinocytosis by preventing actin polymerization.^51,54^ Genistein locally interferes with the actin network and prevents the recruitment of dynamin II, key events in caveolae formation during clathrin-independent endocytosis.^53,55^ Finally, wortmannin is an inhibitor of PI3K kinase, which is involved in pseudopod extension and membrane insertion during phagocytosis.^56,57^ Based on the results described above, 0.05 mg Fe mL^–1^ and 2 h of exposure were selected as the optimal conditions to maximize internalization with the highest preservation of cell viability. Interestingly, all inhibitors diminished the amount of cell debris in NFA-treated cells, except for wortmannin (Figure S15A). If we assume that these cellular debris may be associated with neural cell necrosis triggered by SPION exposure, some of these inhibitors might be then having an impact on blocking the massive internalization of SPIONs. In the case of NFD, this hypothesis is unlikely to occur as cellular debris augmented after incubation with genistein, cythocalasin D, and wortmannin.

We next investigated the SSC^high^ population, hypothesized as the one with the tightest SPION interaction, in the presence of these inhibitors to identify eventual disturbances of SPION uptake (Figure S15B). Surprisingly, we found no remarkable decreases in the SSC values for any condition. Similar findings were observed for FSC values (Figure S15C). Taken together, these results showed an almost negligible blockade of SPION cell uptake except for the impairment of massive internalization of NFA triggering necrosis-induced cellular debris mediated by all inhibitors except for wortmannin. ICP-OES data supported these results, with the cell uptake being similar regardless of the presence of this inhibitor for both samples (54 and 15 pg Fe mL^–1^ for NFA and NFD, respectively, after 24 h of incubation at 0.05 mg Fe mL^–1^). A plausible explanation of these limited effects could be the involvement of diverse mechanisms of SPION entrance in these primary neural cells. It is worth noting that there is a mistaken assumption that pharmacological inhibitors are capable of specifically and completely blocking a single endocytic pathway.^58^ For example, chlorpromazine and cytochalasin D are not efficient in all cell types, while genistein can affect various processes.^58^ Similar mechanisms of cell entry (likely plural) and internalization rates are expected for NFA and NFD samples having similar particle size, shape, and surface coating.^51^ Nonetheless, the efficiency of the uptake of carboxydextran-coated SPIONs by human mesenchymal stem cells has been related to the number of carboxyl groups on their surface.^59^ Specifically, a small quantity of carboxyl groups (i.e., negative charges) seemed already sufficient to induce cell uptake, with a further increase in their density causing a decreased internalization. This might be the case of our SPIONs, NFA being more efficiently internalized by these neural cells than NFD due to their slightly less negative surface charge (see values in Table 1) and therefore a smaller protein corona.

**Figure 4:**
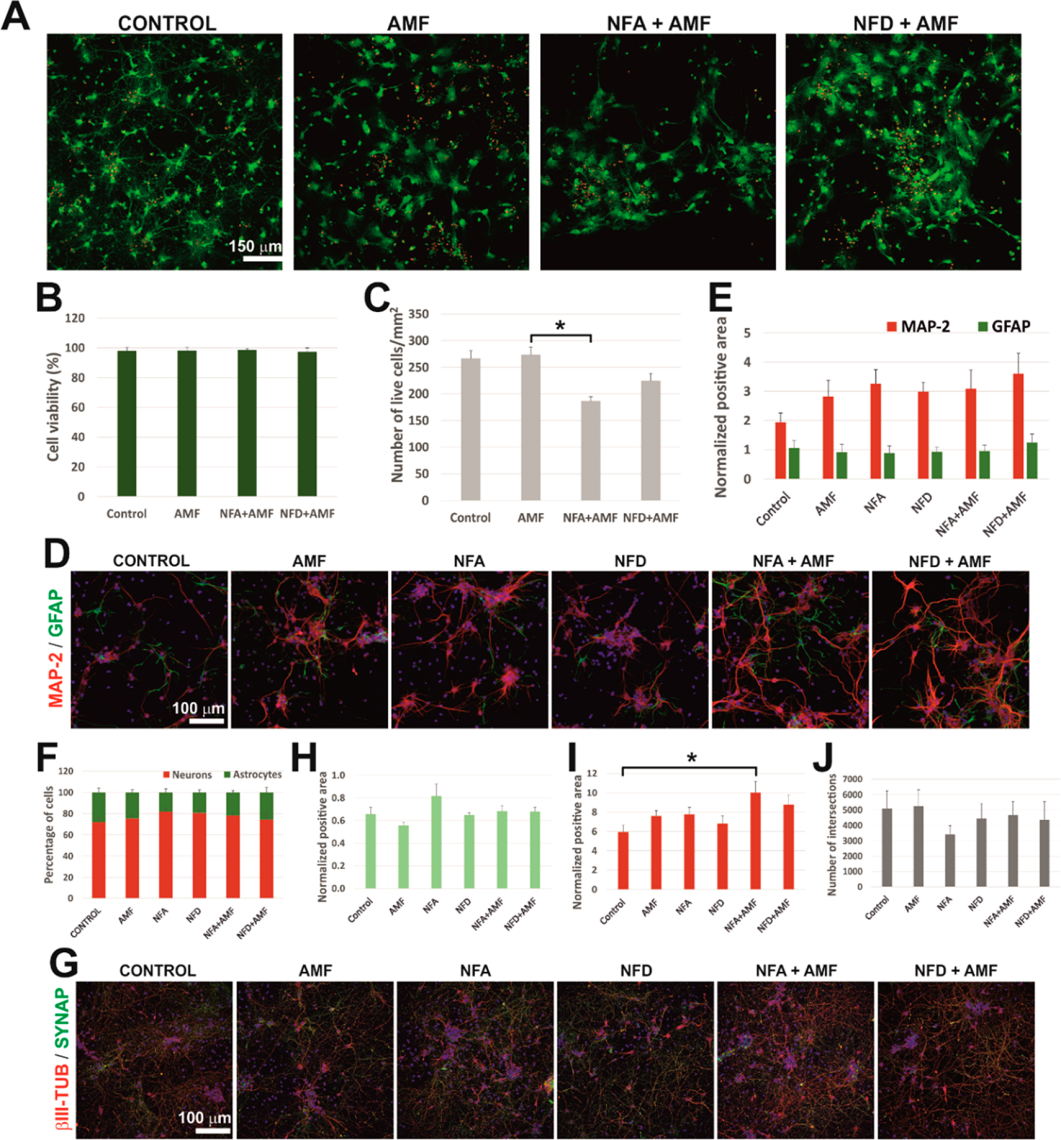
Impact of the application of an alternating magnetic field (AMF) on primary neural cell viability, differentiation, and synapse formation in the presence of NFA and NFD SPIONs. (A) Representative CLSM images illustrating live (green) and dead (red) cells under the different conditions tested. Cell viability expressed as (B) percentage and (C) number of live cells per mm^2^. (D) Representative CLSM images illustrating neurons (MAP-2^+^ cells, red), glial cells (GFAP^+^ cells, green), and cell nuclei (Hoechst, blue). (E) Normalized positive area for MAP-2 and GFAP. (F) Number of positive cells for MAP-2 and GFAP expressed as a percentage of the total number of cells. (G) Representative CLSM images illustrating neurons (βIII-tubulin^+^ cells, red), synapsis (synapthophysin^+^ elements, green), and cell nuclei (Hoechst, blue). (I) Normalized positive area was observed for synaptophysin (H) and β-III tubulin. (J) Number of intersections of the βIII-tubulin neurite arborization. Statistical significance: *p < 0.05.

In order to prove the differential capacity of these SPIONs to drive magnetic actuation, we next explored the application of an alternating magnetic field (AMF). Cell viability was preserved after AMF application, regardless of SPION concentration and type (Figure 4A, B). Nonetheless, a significant reduction in the number of live cells per area unit was found when NFA and AMF were combined (*p* = 0.041* with respect to AMF; Figure 4C). Neural differentiation was then studied. Highly interconnected cultures were formed in all conditions (Figure 4D), without affectation of their differentiation patterns by AMF application when measured as positive area (Figure 4E; *p* = 0.342 for MAP-2 and *p* = 0.913 for GFAP) and number of positive cells for each phenotype (Figure 4F; *p* = 0.28). When identifying mature synapses (Figure 4G), none of the treatments, either alone or in combination, affected the amount of synaptophysin detected (*p* = 0.57; Figure 4H). Moreover, the combined application of NFA and AMF enhanced β-III tubulin arborization (Figure 4I; *p* = 0.016 with respect to the control), although the amount of intersections for this β-III tubulin was not significantly affected by any of the conditions tested (Figure 4J; *p* = 0.819). Overall, magnetic stimulation in the presence of both SPIONs preserved the formation of highly viable neural networks with active synapses. NFA SPIONs triggered a slightly superior impact of AFM application than NFD, as expected from their higher magnetic response, by decreasing the number of live cells but increasing the abundance of β-III tubulin. In line with these results, Semeano et al. reported that the exposure of murine embryonic and human induced pluripotent stem cells to magnetite nanoparticles in the presence of a static magnetic field favored the induction of neural lineage differentiation.^60^ The fact that neurons, which are metabolically less resistant than astrocytes (GFAP^+^ cells),^61^ are preserved under these conditions reinforces the attractiveness of these magnetic nanomedicines for future therapeutic use. Indeed, their potential could be even higher if combined with biocompatible scaffolds, as reported for SPION-doped poly(l-lactide) (PLLA) fibers in combination with an AMF.^62^

**Figure 5:**
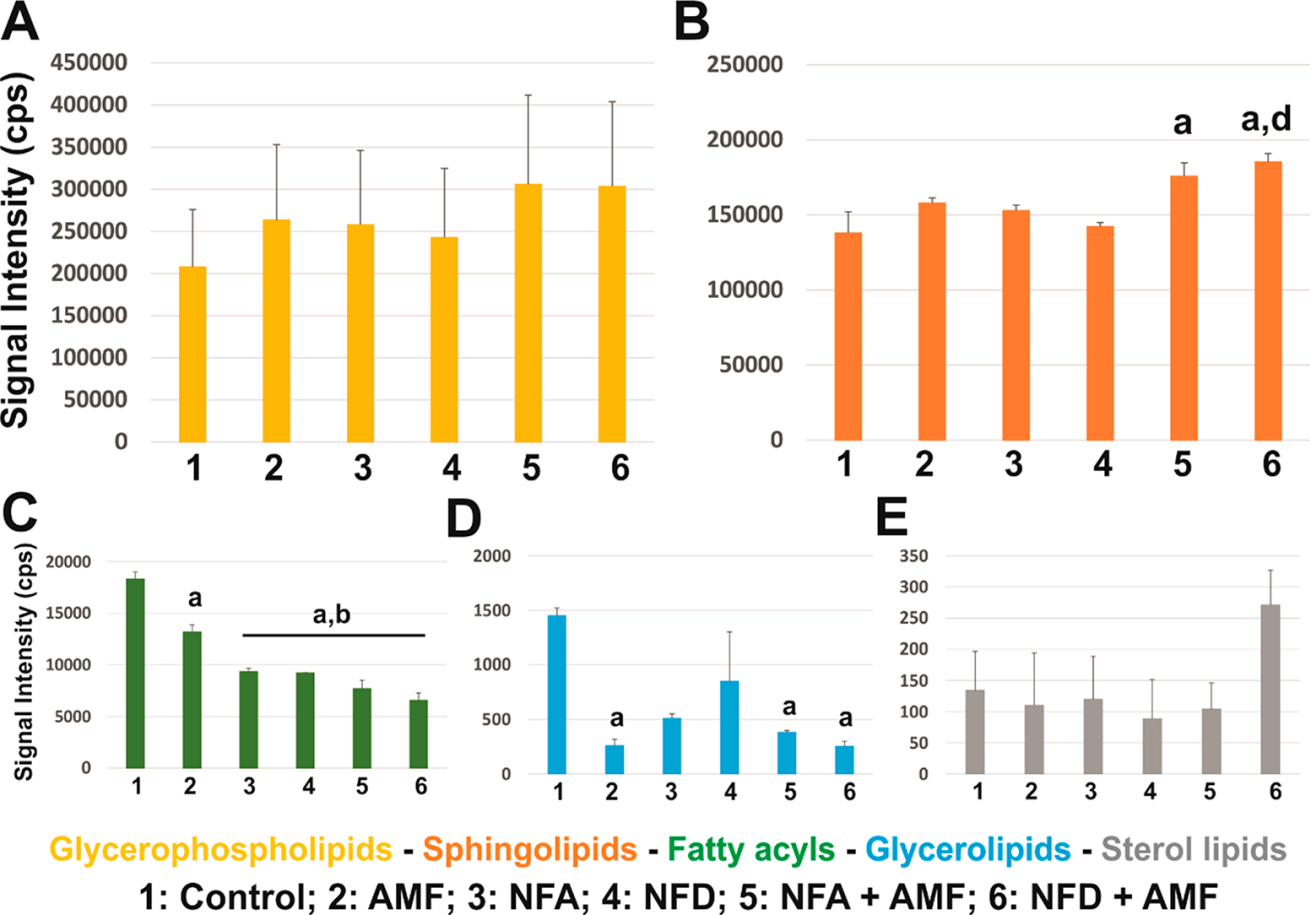
Relative contribution of the main lipid structural categories of the lipidome in primary neural cell cultures after the different conditions tested expressed as signal intensity (cps) for: (A) glycerophospholipids, (B) sphingolipids, (C) fatty acyls, (D) glycerolipids, and (E) sterol lipids. Statistical significance: *p < 0.05 with respect to control (a), AMF (b), and NFD (d). Further details on specific lipid classes are provided in the Supporting Information (Table S2).

The functioning of neural cells and tissues is largely dependent on lipids, which play fundamental roles in the formation of cell membranes, intracellular signaling, and energy production.^63^ They also display pivotal structural functions, as neural cell membranes form largely arborized and intricate networks.^64^ In this context, the effects of SPIONs on the neural cell lipidome remain unknown. To bring some insights into this matter, we next studied the ENPC lipidome after exposure to NFA and NFD SPIONs in the presence of magnetic actuation. From the more than 1,000 lipid species identified by shotgun lipidomics, we focused on the 10 most abundant lipid classes found (Table S2). The relative lipid abundance found agrees with previously reported data for the lipidome of mouse neural cells and tissues,^65^ which also found phosphatidylcholine (PC), phosphatidylethanolamine (PE), and cholesterol within the most abundant lipids in a wide range of neural cell populations and brain regions. The brain cortex origin of our primary neural cells was also consistent with results by Fitzner et al.,^65^ who indicated that both the prefrontal and motor cortices are characterized by a higher proportion of PC, phosphatidylserine (PS), sphingomyelin (SM), and ceramides (Cer). When plotted by categories, differences in the lipidic profiles were observed among the treatment groups. First, glycerophospholipids, the most abundant lipids in all conditions, showed a noticeable, but not significant, trend of increase after SPION exposure and/or AMF application (Figure 5A). For this lipid category, a synergistic effect was found for SPION-treated cells with AMF, expected to result from their response to magnetic stimulation. This same trend reached statistical significance for sphingolipids (Figure 5B; NFA+AMF vs control: *p* = 0.026*; NFD+AMF vs control: *p* = 0.005**; NFD+AMF vs NFD: *p* = 0.011*), with a superior impact for NFD over NFA SPIONs. Contrarily, both fatty acyls (Figure 5C; *p* < 0.001*** for all significant comparisons) and glycerolipids (Figure 5D; NFA+AMF vs control: *p* = 0.047*; NFD+AMF vs control: *p* = 0.023*) dramatically decreased after SPION exposure and/or AMF application. Finally, sterol lipids remained similar to control samples except for the combination of NFD with AMF, in which augmented (difference not statistically significant, *p* = 0.671).

In more detail (Table S2), SPION exposure generally augmented the major lipids PC, PE, and SM, although statistical significance was only reached for the combined treatment with AMF (PC and SM: both NFA and NFD; PE: NFD). PS was significantly augmented only after exposure to AMF in combination with NFD SPIONs. Neither phosphatidic acid (PA), phosphatidylinositol (PI), nor Cer was significantly varied by SPIONs exposure or AMF application. Contrarily, free fatty acids and triacylglycerides (TAG; esters of glycerol with three fatty acids) dramatically decreased after SPION exposure, with a clear synergistic effect driven by the combination of SPION with AMF. For both lipid classes, the exposure to NFD SPIONs under magnetic stimulation, but not alone, mediated the most substantial differences in cell lipids. Finally, cholesterol ester was significantly reduced after the NFD exposure and AMF application. Interestingly, while NFA magnetically stimulated by AMF diminished cholesterol, NFD with AMF had the completely opposite effect. These significant variations in the neural cell lipidome corroborate the close and distinctive interactions of both types of SPIONs with cellular lipids, which are mostly located in their membranes. Specifically, NFD SPIONs prompted more dramatic actions at the lipid components of the cell. Generally, these effects were boosted by the combined application of an AMF. This specific lipid targeting may be related to a more predominant location of NFD SPIONs at the cell membrane, rather than internalized, with respect to NFA (as corroborated by ICP-OES) or a tighter interaction with its lipids, which compensates for their inferior magnetic response. The slightly higher negative surface charge of NFD SPIONs might be at the density limit for this type of SPIONs that starts conflicting internalization by negative charges repulsion mechanisms, in agreement with previous findings.^59^ Recently, microRNA-coated nanoparticles with a similar size (70 nm) to those described herein and a negative coating (−22 mV) have shown improved systemic plaque delivery for alleviating atherosclerosis. In one such study, the authors claim that the negative surface charge alone does not guarantee improved plaque delivery.^66^

Overall, SPION exposure and AMF application augmented lipid classes with unique structural and biological functions in cell membranes such as glycerophospholipids and sphingolipids, often located within raft nanodomains in cell membranes and closely interacting with cholesterol and proteins. An especially interesting example is the SM, known to form a stable and chemically resistant outer leaflet of the plasma membrane lipid bilayer. Importantly, SM has been pointed out as essential for the internalization of transferrin and thence of iron into cells and for the activity of many membrane-bound proteins, including ion channels and receptors. The involvement of this lipid in iron metabolism supports the significant increase found in neural cells exposed to both types of SPIONs and AMF. On the contrary, those lipids related to energy storage and production as fatty acyls and glycerolipids significantly decreased after any treatment. Even with a small role in neuronal lipid metabolism, they are the storage form of lipid precursors in neurons.^67^ Their substantial decrease in neural cells exposed to SPIONs or AMF might be indicative of a larger cell consumption of these energy stores under these treatment conditions due to the activation of stress cell routes. Nonetheless, the complexity of these interactions requires further studies to fully unravel the mechanisms behind these specific SPION actions on lipid components and their functional and therapeutic impact.

**Figure 6:**
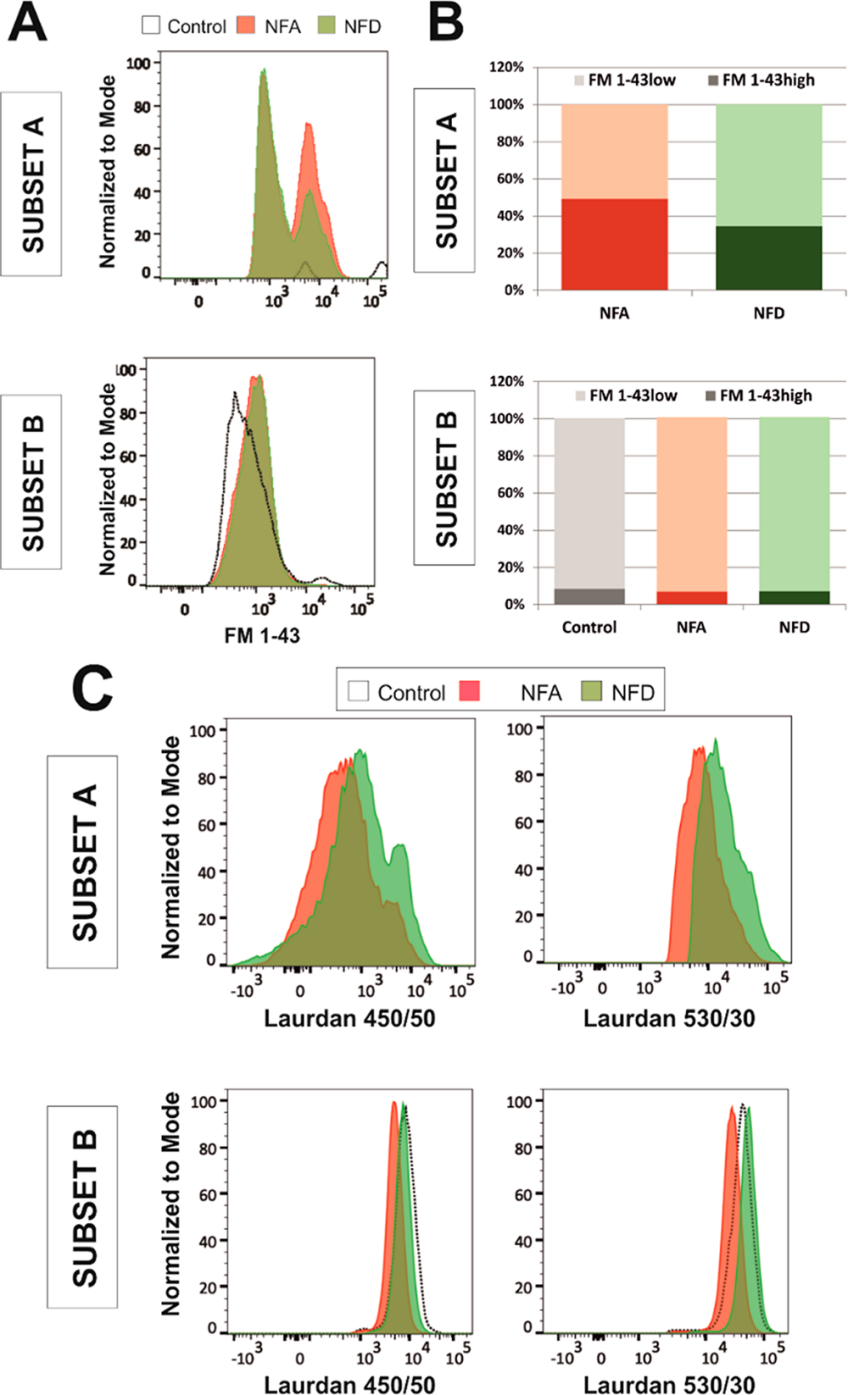
Impact of SPION physicochemical properties on the lipid components and membrane functioning of primary neural cells. Cell membrane studies were performed by using the FM1-43 probe and corresponding results for subsets A (top) and B (bottom), expressed as (A) histogram overlays and (B) cell percentages. (C) Cell membrane studies using the Laurdan probe and corresponding results for subsets A and B, expressed as histogram overlays at both fluorescence ranges (450/50 for the gel state and 530/30 for the liquid state).

Based on the significant and distinctive impact of both SPIONs on pivotal lipids, we used two lipophilic probes, FM1-43 and Laurdan, to further analyze the impact of NFA and NFD SPIONs on cell membrane function, including integrity and fluidity. Particularly, FM1-43 is a lipophilic styryl compound used in studies of the plasma membrane and vesiculation, including endocytosis and exocytosis processes.^68^ As a water-soluble dye, FM1-43 is believed to insert into the outer leaflet of the surface membrane, where it becomes intensely fluorescent. By using flow cytometry, we clearly identified three cell populations, FSC^dim^, subset A, and subset B, with two distinctive MFI values, FM1-43^high^ and FM1-43^low^ (Figure S2). The first set of cells, identified as FSC^dim^, was present in the control cells and both types of SPION-treated cells. Their larger percentage of FM1-43^high^ events and higher MFI value agreed with a poorer integrity of these events (Figure S16), so they were assigned to damaged cells and cell debris and then discarded from further analyses. Contrarily, subset A only appeared in the presence of SPIONs (Figure 6A, top, and Figure S17), in accordance with the previous assumption of those cells more closely interacting with SPIONs (earlier named as SSC^high^). Interestingly, there was a significantly larger population of FM1-43^high^ cells in NFA-treated samples (49.3%) than NFD-treated samples (34.6%) (Figure 6B, top), again confirming a higher capacity of NFA SPIONs to interact with these primary neural cells. The similarity in MFI values between both types of SPIONs seems to support similar cell membrane integrity and probe internalization (Figure S18A). When focused on subset B (Figure 6A, bottom and Figure S17), cells treated with both SPIONs behaved comparably to control cells in terms of percentages for FM1-43^high^ and FM1-43^low^ populations (Figure 6B, bottom). However, the exposure to SPIONs, regardless of the type, decreased the MFI of FM1-43^high^ cells and increased that of FM1-43^low^ cells (Figure S18B). As most B-gated cells corresponded to FM1-43^low^ (>90% for all conditions), the increased MFI value in SPION-treated cells is likely indicating a superior internalization of the probe with respect to control cells. This probe internalization might be mediated by the generation of hydrophilic pores at the plasmatic membrane, as previously proven in simulations for strongly charged nanoparticles with a size comparable to membrane thickness^69,70^ and/or the stimulation of internalization pathways in the presence of SPIONs. Based on MFI values, FM1-43^low^ cells in subsets A and B likely belong to a similar subpopulation that remained closer to control cells. Further elevation of MFI values for FM1-43^high^ cells from subset A (6618 au for NFA and 6600 au for NFD) with respect to those for FM1-43^low^ cells in subsets A and B (∼900 au for all conditions) indicates a clear alteration of the cell membrane integrity as a result of the interaction with SPIONs. Indeed, NFA SPIONs were able to interact with a larger cell population than NFD, although the magnitude of their impact at the membrane seemed similar for both.

We next used the Laurdan dye to deepen the correlation of these effects with cell membrane fluidity. As for FM1-43, we could clearly identify the three same (Figure S3). Regarding subset A (again absent in control cells), both fluorescence emissions at 450/50 and 530/30 were enhanced in NFD-treated cells with respect to those in NFA-treated ones (Figure 6C, top). Corresponding MFI values followed the same trend (Figure S18C). For subset B (present in the three groups), NFD-treated cells also showed higher values than NFA-treated cells for both wavelengths (Figure 6C, bottom; Figure S18D). All of these variations in fluorescence could be more easily observed when plotted together (Figure S18E). When quantified as the ^405^GP_ex_, cells in subset B showed more negative general polarization values when treated with SPIONs (−0.70 for NFA and −0.75 for NFD) than on control conditions (−0.66). Those cells more closely interacting with SPIONs (subset A) displayed even more negative values (−0.88 and −0.89 for NFA and NFD SPIONs, respectively). Based on this, both types of SPIONs significantly increased cell membrane fluidity with respect to control cells. Importantly, the impact of NFD was superior to that of NFA as expected from their more dramatic effects on cell lipids related to their slightly larger surface area for molecular interactions and their more negative surface charge hampering their uptake with respect to NFA. This impact in cell membrane fluidity correlates well with the significant increase in PC and SM and decrease in cholesterol found in SPION-treated cells (again, larger for NFD than NFA SPIONs), all three structural lipids at the neural cell membrane. This fluidization of the cell membrane mediated by SPIONs is of pivotal importance when designing their use as therapeutic nanomedicines.

**Figure 7:**
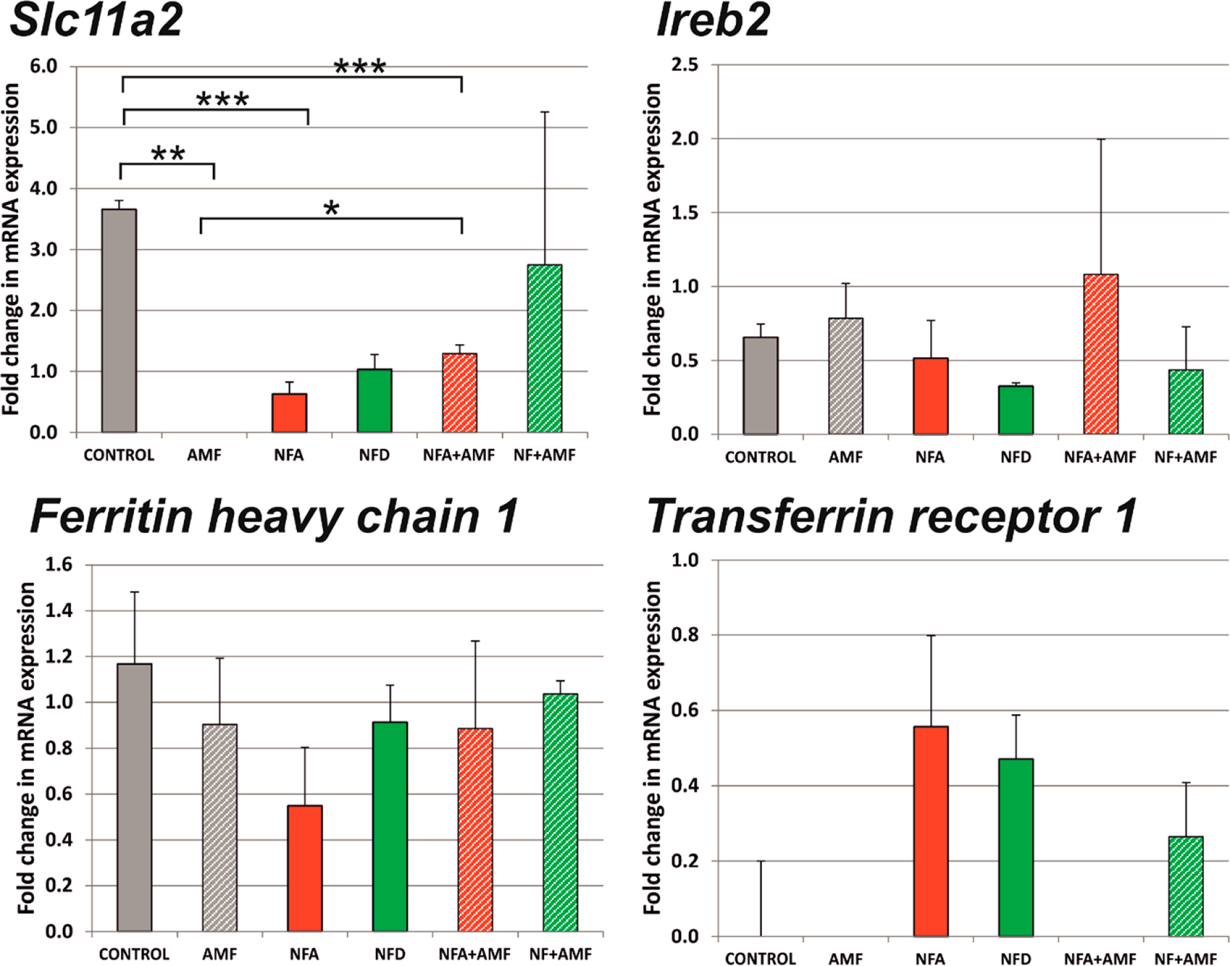
Impact of the exposure to SPIONs and the application of an alternating magnetic field (AMF) in the mRNA expression of specific genes related to iron metabolism (*Slc11a2*, *Ireb2*, *Fth1*, and *Tfrc-1*) in primary neural cells. Statistics: *p < 0.05, **p < 0.01, and ***p < 0.005.

Finally, we explored the impact of the physicochemical properties of these two SPIONs on the mRNA expression of four selected genes related to iron metabolism (*Slc11a2*, *Ireb2*, *Fth1*, and *Tfrc*). First, it is worth noting that the concentration, quality, and integrity of the mRNA of all samples were optimal (RNA integrity number, RIN ≥ 9.7). Nonetheless, most of the genes analyzed were detected late (PCR cycle ≥18.8, mean PCR cycle of detection from all samples and genes analyzed = 29.9), but within the 20–30 cycle range previously reported for other genes in RT-qPCR studies.^71^Figure 7 illustrates mRNA expression in fold change (normalized by the putative gene *18S* as described by others^72^). From the four genes analyzed, *Slc11a2* (i.e., solute carrier family 11 member 2) was the gene with the most dramatic modifications, which included a significant reduction under all treatment conditions except for the combination of NFD with AMF. Neither *Ireb2* (i.e., iron responsive element binding protein 2) nor *Fth1* (i.e., ferritin heavy chain 1) showed significant changes in their mRNA expression in any of the conditions tested. Interestingly, the mRNA of *Tfrc* (i.e., transferrin receptor) was detected in SPION-treated cells without AMF, regardless of SPION type (*p* = 0.137), but had a negligible expression in control and AMF alone conditions. This finding may be related to a higher demand of the transferrin receptor in these cells due to the massive presence of iron, as the transferrin receptor 1 is known to respond to iron concentrations. Furthermore, it agrees with the significant increase in SM found in SPION-treated cells, which is a lipid involved in the internalization of transferrin. Taken together, the slight differences in the physicochemical properties of SPIONs showed a minor impact in the mRNA expression of the selected genes related to iron metabolism. Nonetheless, the exposure to SPIONs with and without the application of an AMF had significant effects on the expression of certain genes, such as *Slc11a2* and *Tfrc-1*. Further studies are necessary to elucidate the impact of SPIONs on the specific transcription factors and routes that govern the expression of these genes.

## 4 Conclusions

We have identified specific responses associated with SPION type, concentration, and exposure time and AMF application in primary neural cells. Both NFA and NFD SPIONs were found at the cell membrane and intracellularly without a clear and unique internalization route identified. NFA SPIONs (i.e., those with a denser multicore structure that resulted in a lower surface area, a slightly less negative surface charge, and a higher magnetic response) had a superior capacity to be internalized and impact cell viability and complexity. Both SPIONs dramatically augmented lipids such as PC, PE, and SM, while reducing free fatty acyls and TAG. These lipid changes were associated with an enhancement of cell membrane fluidity. For these lipid-associated effects, NFD SPIONs (i.e., those with a less compact multicore structure resulting in a larger surface area and a slightly more negative surface charge) were clearly superior, likely related to a more preferential membranal location and/or a tighter interaction with lipids at the cell membrane than NFA, corroborated by their lower cell uptake. Contrarily to lipids, the physicochemical differences between these two SPIONs had a minor impact on the mRNA expression of selected genes related to iron metabolism. The capacity of these SPIONs to differentially modulate the neural cell fate and function by slight changes in their physicochemical properties encourages their exploration as lipid-targetable magnetic nanomedicines in the context of neural diseases. An important challenge that remains is the segregation of the surface area and surface charge in negatively charged SPIONs to unravel the specific contribution of each feature in the cellular and molecular responses found.

## Data Availability

All data are available in the main text or the Supporting Information. Additional raw and processed data required to reproduce these findings will be available to download from DIGITAL.CSIC upon acceptance or from the authors upon request.

## Supporting Information Available

Extended version of the Material and Methods; gating and analysis strategy for flow cytometry studies and specific studies with FM1-43 and Laurdan probes; basic structural, colloidal and magnetic characterization of SPIONs (i.e., TEM, size distribution, DLS distribution, ZFC/FC, heating curves under AMF, XRD, SEM images, EDX spectra, and FTIR spectra over the 4000–500 cm^–1^ range); dynamic light scattering measurements of SPIONs incubated in Neurobasal media; representative CLSM images of viability studies with SPIONs; representative SEM images of the detailed surface interaction of SPIONs and ENPCs; representative TEM images of SPION-treated ENPCs under bright and dark field conditions, control cells, and SPIONs; percentage of cell debris for all SPION incubation conditions; histograms illustrating viability results by flow cytometry for all SPION incubation conditions; SCC/FSC dot plots from flow cytometry studies with ENPCs and L929 fibroblasts; histogram overlays of FSC values from SPION-exposed ENPCs; histogram overlays of SSC and FSC values for ENPCs exposed to SPIONs in the presence of inhibitors; percentage and MFI values of FSC^dim^ subset in SPION-treated ENPCs; FM1-43/FSC dot plots for ENPCs incubated with SPIONs; MFI values for FM 1-43 and MFI and dot plots for Laurdan; Table S1 containing primer sequences used in RT-qPCR studies; Table S2 summarizing main lipid classes in the different treatment groups and corresponding statistics (PDF)
