# Supplementary material for: Cellular and Molecular Processes Are Differently Influenced in Primary Neural Cells by Slight Changes in the Physicochemical Properties of Multicore Magnetic Nanoparticles
Source: ACS Appl Mater Interfaces. 2023 Mar 28;15(14):17726–41. doi: 10.1021/acsami.3c02729 (PMC10103129; doi:10.1021/acsami.3c02729)
Supplement: Supplementary file 1 — am3c02729_si_001.pdf [file am3c02729_si_001.pdf]

Supporting Information

Cellular and molecular processes are differently influenced in primary neural cells by slight changes in the physico-chemical properties of multicore magnetic nanoparticles

Esther Benayas,[1] Ana Espinosa,[1] M. Teresa Portolés,[2,3] Virginia Vila-del Sol,[4] M. Puerto Morales,[1] and María C. Serrano[1,]*

[1] Instituto de Ciencia de Materiales de Madrid, Consejo Superior de Investigaciones Científicas, calle Sor Juana Inés de la Cruz 3, 28049-Madrid, Spain. E-mail: mc.terradas@csic.es

[2] Departamento de Bioquímica y Biología Molecular, Facultad de Ciencias Químicas, Universidad Complutense de Madrid, Instituto de Investigación Sanitaria del Hospital Clínico San Carlos (IdISSC), 28040 Madrid, Spain

[3] CIBER de Bioingeniería, Biomateriales y Nanomedicina, CIBER-BBN, ISCIII, 28040 Madrid, Spain

[4] Hospital Nacional de Parapléjicos, SESCAM, Finca de la Peraleda s/n, 45071 Toledo, Spain

Extended Version of Material and Methods

1. Material

Iron salts, such as ferric chloride hexahydrate (98 %), ferrous chloride tetrahydrate (98 %) and ferric nitrate nonahydrate (98 %), sodium hydroxide (98 %), N-methyldiethanolamine (NMDEA, 99 %), diethylene glycol (DEG, 99 %), ethyl acetate (99.8 %), meso-2,3-dimercaptosuccinic acid (DMSA, 98 %), nitric acid (65 %), hydrogen peroxide (30 wt%) and reagents for sodium phosphate buffers were purchased from Sigma-Aldrich. Ethanol (96 %) was purchased from Scharlau. Other chemical reagents and primary antibodies were purchased from Merck and used as received, unless otherwise indicated. Neurobasal™ media, B-27 supplement, immunofluorescence probes, and secondary antibodies were purchased from Fisher Scientific.

2. Synthesis and characterization of iron oxide nanoparticles

The synthesis of multicore SPIONs was based on previous works.[41,42] Two heating systems were used here, one based on a heating mantle and glass under reflux and mechanical stirring conditions (NFD), and other based on a teflon lined stainless steel autoclave (NFA), which is a closed system that allows to minimize handling. In both cases, 3.2 mmol of $FeCl_3{\cdot}6H_2O$ and 1.6 mmol of $FeCl_2{\cdot}4H_2O$ were dissolved in 64 g of a mixture of DEG and NMDEA at 50 %. Separately, NaOH was dissolved in 32 g of the same DEG and NMDEA mixture under ultrasounds for 30 min. The two separated solutions were mixed for 5 min, transferred to the glass beaker or to the autoclave and heated at 220 ºC during 16 h. The precipitate was recovered using a magnet and washed with a mixture of ethanol and ethyl acetate four times. The nanoparticles were subjected to an acid treatment

following a protocol previously reported.[43] The precipitate was dispersed in nitric acid (10 %). Then, $Fe(NO_3)_3 \cdot 9H_2O$ was added and the mixture was heated to 80 ºC for 45 min. After cooling, the nanoparticles were dispersed with nitric acid (10 %) again. Finally, the sample was washed by magnetic decantation with ethanol and acetone, and finally dispersed in water. Acetone residues were eliminated using a rotatory evaporator.

All synthesized magnetic nanoparticles were coated with citric acid. For this purpose, the pH of the nanoparticle suspension previously prepared containing 20 mg of Fe was adjusted to pH 2 and 13 mL of 0.1 M citric acid were added, heated at 80 °C for 30 min and then, centrifuged, washed with distilled water and dispersed, first at pH 11, and then, at pH 7. Colloidal properties were analyzed by Dynamic Light Scattering (DLS) in a NanoZsizer apparatus from Malvern to determine the hydrodynamic size (D_{hydro}) and surface charge. The core size and morphology of the nanoparticles were analyzed by transmission electron microscopy (TEM, JEOL JEM 1010). Size distribution was determined by measuring ~150 particles with the ImageJ digital software. Data were fitted to a log-normal curve and the mean value (D_{TEM}) obtained. Surface and internal composition was analyzed by energy-dispersive X-ray spectroscopy (EDX) and the spectra were taken on a field emission scanning electron microscope (SEM, FEI Verios 460) at 2 kV accelerating voltage and a probe current of 13 pA. In addition, Fourier-transform infrared spectroscopy (FTIR) was carried out at in a spectrophotometer Bruker Vertex 70V with 2 cm^{-1} resolutions in KBr pellets and the FTIR absorbance spectra were collected over the 4000-500 cm^{-1} range.

Magnetic characterization was carried out using Vibrating Sample Magnetometry (VSM, Oxford instrument) and SQUID magnetometers (Quantum Design). Hysteresis

loops were recorded at room temperature after applying a magnetic field of ± 5 T and saturation magnetization by extrapolation to infinity field (Ms) and coercivity (Hc) were obtained in the VSM. The sample consisted in 100 μL of the colloidal suspension dried on a piece of cotton. Zero Field Cooling and Field Cooling (ZFC/FC) magnetization curves were recorded between 300 and 5 K at 100 Oe in the SQUID. Fe concentration in the colloidal and cell suspensions was determined by inductively coupled plasma optical emission spectroscopy (ICP-OES) in a PERKIN ELMER OPTIMA 2100 DV apparatus after digestion with nitric acid and aqua regia at 90 ºC (1.2 x 10^6 cells in 10 mL). Samples were digested in aqua regia and diluted to a known volume. The heating efficiency was evaluated in a Five Celes apparatus with an Osensa temperature probe, measuring the temperature change under the application of an alternating magnetic field (280 kHz-20 mT and 90 kHz-60 mT) in 1 mL of sample at 20-25 mM Fe concentration. The crystal structure of the sample was identified by X-ray diffraction using a Bruker D8 ADVANCE diffractometer with Cu Kα radiation, between 10º and 90º in 2θ. Crystal size was calculated from the (311) peak broadening.

Dynamic light scattering was used to assess the protein corona formation when NFA and NFD nanoparticles were incubated with fresh (and complete) Neurobasal culture media at different time points (0, 15, 30, 60 and 120 min and 24 h) at 37 ºC in a 5% CO_2 atmosphere (inside a sterile cell incubator).

3. *Primary neural cells isolation, culture and SPIONs exposure*

Embryonic neural progenitor cells (ENPCs) were obtained from cerebral cortices of Wistar rat embryos as previously described.[44] Adult female Wistar rats were provided by

the animal facilities of the National Hospital for Paraplegics and sacrificed when gestation reached 16–17 days (E16-E17). All the experimental protocols for cell collection adhered to the regulations of the European Commission (directives 2010/63/EU and 86/609/EEC) and the Spanish Government (RD53/2013 and ECC/566/2015) for the protection of animals used for scientific purposes. A total of 15 independent cell cultures ($N \geq 3$ per cell assay and triplicates per culture condition) were carried out, with cell viability being always above 85 %. Prior to cell culture, plates were coated with an aqueous solution of poly(L-lysine) (PLL) (45 μg mL^{-1}) for 1 h at room temperature, later washed with sterile water, and finally conditioned for 1-2 h in complete culture medium in a sterile incubator at 37 ºC under a 5% CO_2 atmosphere. Cell seeding density was 25 x 10^3 cells cm^{-2} for all experiments except for qRT-PCR studies in which seeding density was doubled to increase the quantity of mRNA per condition. For flow cytometry studies, ENPC suspensions right after isolation were exposed to SPIONs at a density of 100,000 cells mL^{-1} (a total suspension of 5 mL per treatment condition). Cells were maintained for different time points (days-*in-vitro*, DIV) in complete Neurobasal™ culture medium containing B-27 supplement (2 %), streptomycin (100 UI mL^{-1}), penicillin (100 UI ml^{-1}), and GlutaMAX (1 %), which was half-replaced every 3-4 days. At different culture times as specifically indicated for each type of assay, cells were exposed to either NFA or NFD SPIONs at the different concentrations of selection in a sterile incubator at 37 ºC under a 5% CO_2 atmosphere. After 24h of exposure, SPION-exposed cells were gently washed with phosphate buffer saline (PBS) twice and maintained in fresh culture media for another 24 h.

4. Magnetic stimulation studies

For magnetic stimulation, cell culture Petri dishes of 3.5 cm in diameter were used. Prior to ENPC culture, Petri dishes were coated with PLL as described above. Cells were then seeded and maintained in culture for different time points depending on the tests to be carried out. A total of six different conditions were evaluated: (1) cells without SPIONs nor magnetic field (Control), (2) cells only exposed to an alternating magnetic field (AMF), (3) cells only exposed to NFA SPIONs (NFA), (4) cells only exposed to NFD SPIONs (NFD), (5) cells exposed to both NFA SPIONs and a magnetic field (NFA + AMF) and (6) cells exposed to both NFD SPIONs and a magnetic field (NFD + AMF). ENPC cultures were exposed to the selected concentration/s of either NFA or NFD SPIONs 48 h before the application of the magnetic field. The hyperthermia treatment of selection was an alternating magnetic field of 20 mT for 60 min, with a frequency of 280 kHz, applied by using a Five Celes equipment.

5. *Morphological studies by electron microscopies*

Cell culture morphology after the exposure to SPIONs was first studied by field-emission scanning electron microscopy (FESEM). Samples were fixed with glutaraldehyde (2.5 % in PBS) for 45 min. After gentle washing in distilled water, dehydration was performed by using series of ethanol solutions for 15 min (2 washes) and a final dehydration in absolute ethanol for 30 min. Samples were then dried at room temperature for at least 48 h. After mounting in stubs and coating with a nanometer-thick Chromium layer under vacuum, the morphology of the samples was immediately after characterized by using a field-emission Philips XL30 S-FEG microscope.

SPIONs internalization in ENPCs was characterized both in culture and suspension by transmission electron microscopy (TEM). For the visualization of SPIONs internalized by cells in culture, ENPCs were seeded on Permanox chambers (Nunc^{TM} Lab-Tek^{TM}) for 14 DIV. At 12 DIV, cells were treated with either NFA or NFD SPIONs at different concentrations. Samples were then fixed with a mixture of paraformaldehyde (PFA) 4% and glutaraldehyde 1% in 0.1 M phosphate buffer for 1 h and then post-fixed in osmic tetroxide (1% in distilled water) for an additional hour. Next, dehydration was carried out by immersion in successive solutions of ethanol at increasing concentrations (30 %, 50 %, 70 %, 95 % and 100 %), with a final step in pure acetone. Samples were included then in the resin Durcupán by consecutive immersion steps at increasing concentrations (1:3, 1:1 and 3:1 in acetone). The final samples in pure resin were then polymerized at 60 ºC for 48 h. Ultrathin sections (~ 60 nm) were obtained and subsequently stained with uracil acetate and lead citrate. The visualization was carried out by using a Jeol JEM 1010 microscope (Japan) at 80 kV with a coupled camera (Gatan SC200, USA) for image acquisition.

For the visualization of SPION uptake by cells in suspension, ENPCs right after isolation were exposed to either NFA or NFD at different concentrations for 4 h at 37 °C. Cell suspensions were then centrifuged at 300 g for 4 min. The obtained pellet of cells was fixed at room temperature with 4 % PFA and 2 % glutaraldehyde in 0.1 M phosphate buffer at pH 7.4 for 2 h, embedded in a gelatin matrix (10 % in bi-distilled water) and kept on ice until gelatin solidified. Then, it was cut in small cubes to proceed with embedding in epoxy resin as for a tissue block. Post-fixation was carried out with 1 % OsO_4 and 0.8 % $K_3Fe(CN)_6$ in water at 4 °C for 1 h. Samples were dehydrated with ethanol and embedded in epoxy TAAB 812 resin (Laboratories, Berkshire, England) according to standard

procedures. After polymerization, 80-nm-thick (ultrathin) sections were obtained and stained with 2 % uranyl acetate solution in water and Reynolds lead citrate and examined at 100 kV in a Jeol JEM 1400 Flash (Tokyo, Japan) microscope. Pictures were taken with an OneView (Gatan) digital CMOS camera (4K x 4K). Images were acquired in both bright and dark field modes to clearly identify SPIONs inside the cells.

6. Viability studies by confocal fluorescence microscopy

Cell viability in culture was analyzed using a Live/Dead® viability kit according to manufacturer's instructions (Life Technologies). Briefly, the live and dead cells were stained with calcein and ethidium homodimer-1 (EthD-1), respectively. After staining, samples were visualized by using a Leica SP5 confocal laser scanning microscope. The fluorescence of both probes was excited using an argon laser tuned to 488 nm. After excitation, emitted fluorescence was separated by using a triple dichroic filter 488/561/633 and measured at 505-570 nm for green fluorescence (calcein) and 630-750 nm for red fluorescence (EthD-1). Collected images ($n \geq 5$ per condition) were analyzed using the ImageJ software to quantify the area occupied by those positively stained for each marker with respect to the total image area.

7. Viability and internalization studies by flow cytometry

First, flow cytometry studies were carried out to analyze cell viability and the effect of SPIONs internalization in cell size and complexity in ENPC suspensions. Conditions investigated included: (1) cells without SPIONs (control), (2) cells exposed to NFA SPIONs at 4 different concentrations (0.001, 0.01, 0.025 and 0.05 mg Fe mL^{-1}), and (3) cells exposed to NFD SPIONs at 4 different concentrations (0.001, 0.01, 0.025 and 0.05 mg

Fe mL^{-1}). Exposure times were 1, 2, 4 and 24 h in a sterile incubator at 37 ºC under a CO_2 atmosphere (5 %). Briefly, ENPC suspensions right after isolation were exposed to the different treatments in 15 mL Falcon® tubes (5 mL). After the corresponding incubation times, cells were centrifuged at 300 g for 4 min, the supernatants were discarded, and the pellets were suspended in fresh culture media (1 mL). Subsequently, samples were filtered using 12 x 75 mm tubes with a 35 µm nylon cell strainer cap (Falcon) in order to remove cell aggregates and centrifuged again at 300 g at 4 ºC for 4 min. Supernatants were discarded and cellular pellets suspended in 100 µL of ice-cold Annexin V Binding Buffer 1x for staining with Annexin V-FITC and 7-amino-actinomycin D (7-AAD) following the manufacturer instructions (Beckman Coulter Life Sciences). Samples were analyzed on a FACS Canto II cytometer (BD Biosciences) within 30 min after staining and recorded for 2 min with at least 10,000 events recorded in the FSC gate. Flow cytometry data analysis was carried out with the FlowJo 10.7 software (BD Biosciences) following the gating strategy depicted in **Figure S1**. In detail, after cellular aggregates exclusion (A), FSC gate was plotted on an FSC vs SSC dot-plot to remove cellular debris (B). Then, FSC-gated events were analyzed for Annexin V and 7-AAD staining (C). Three subpopulations were identified: (1) Early apoptosis, with events Annexin V^+/7-AAD^-; (2) Late apoptosis, with events Annexin V^+/7-AAD^+; and (3) Live cells which were double negative cells (Annexin V^-/7-AAD^-). The subpopulation characterized by low FSC and high SSC scattering was further gated into FSC gate as FSC^{low} region (D). Subsequently, Annexin V/7-AAD staining was analyzed in the FSC^{low} subpopulation in the same way as the FSC-gated population (E). Finally, changes induced by SPIONs treatment in the SSC intensity of FSC^{low}-gated events were monitored by plotting them in a histogram (F). The SSC high interval was defined by using the corresponding control samples without nanoparticles.

In order to unravel the cellular route for SPION uptake in this cell type, ENPC suspensions were pre-incubated for 2h in a sterile incubator at 37 ºC under a CO_2 atmosphere (5 %) with the following inhibitors covering the most common entry pathways: (i) chlorpromazine to affect clathrin-dependent endocytosis (5 µg mL^{-1}), (ii) amiloride to block macropinocytosis, specifically affecting the sodium-proton exchange (0.5 µg mL^{-1}), (iii) cytochalasin D to influence macropinocytosis by acting on actin polymerization (2 µM), (iv) genistein to affect clathrin-independent endocytosis (5 µM), and (v) wortmannin to inhibit PIK3 and other kinases during phagocytosis (5 µg mL^{-1}). After inhibitors pre-incubation, cell suspensions were then exposed to 0.05 mg mL^{-1} of either NFA or NFD SPIONs for 2 h. Cells without either blocking agents or SPIONs, cells incubated with the different inhibitors in the absence of SPIONs and cells only exposed to the two types of SPIONs (without inhibitors) served as control samples. After corresponding incubations, cell suspensions were prepared for flow cytometry analyses as described above. To corroborate that the incubation conditions were harmless for these primary neural cells, duplicate samples of control cells without either blocking agents or SPIONs and cells exposed to NFA and NFD SPIONs without inhibitors were seeded into PLL-coated petri dishes right after this 4h-treatment. Cell viability and neural differentiation was analyzed after 7 DIV by confocal fluorescence microscopy as described in other sections.

8. *Neural differentiation by confocal fluorescence microscopy*

An immune-labeling procedure was used to investigate the impact of SPIONs exposure on neural cell differentiation at different time points in culture. Briefly, cells were fixed with 4 % PFA in PBS for 10 min at room temperature, rinsed with PBS, and then permeabilized with saponin (0.25 % in PBS + 10 % of fetal bovine serum) for 10 min. The

following primary antibodies were selected: MAP-2 and β-III tubulin for labeling neurons, vimentin for labeling non-neuronal cells including glia and glial fibrillary acidic protein (GFAP) for specific targeting of astrocytes. Additionally, synaptophysin, the most abundant protein in the membrane of synaptic vesicles, was used to visualize synapses in neurons. The secondary antibodies used were Alexa Fluor® 488 anti-mouse in goat IgG (H + L) and Alexa Fluor® 594 anti-rabbit in goat IgG (H + L). Both primary and secondary antibodies were dissolved in PBS containing saponin (0.25 %) to guarantee cell permeability and fetal goat serum (2 %) with the purpose of blocking non-specific bindings. Both primary and secondary antibodies were incubated for 1 h at room temperature in darkness. Finally, samples were washed with PBS three times and cell nuclei were stained with a Hoechst solution for 10 min. Samples were visualized by using a Leica TCS SP5 microscope. The fluorescence of the different fluorochromes was excited and measured as follows: Alexa Fluor® 488 excitation at 488 nm with an argon laser and detection at 507-576 nm, Alexa Fluor® 594 excitation at 594 nm with a helium-neon laser and detection at 625-689 nm and Hoechst excitation at 405 nm with a diode UV laser and detection at 423–476 nm. Capture conditions in each case were stablished by using appropriate positive and negative controls and maintained during the acquisition of all the images. Collected images ($N \geq 5$ per condition) were analyzed using the ImageJ software. Both the area and the number of cells positively stained for each marker was quantified and normalized by cell density (obtained from Hoechst images).

9. *Lipidome studies*

ENPC cultures on petri dishes (3.5 cm in diameter) were exposed to the different conditions and then trypsinized. Cell suspensions were centrifuged at 19,900 rpm at 4 ºC

for 15 min. NaCl (0.88 %, 100µL, at 4 ºC), EquiSPLASH internal standard (10 µL) and chloroform/methanol (2:1; 500 µL) were added to the pellets. Suspensions were then incubated at – 40 ºC for 15 min and sonicated 30 s at 80 % with a UP50H homogenizer (5 times) a -40ºC. These last two steps were repeated three times. Samples were centrifuged at 14,000 rpm for 15 min at 4 ºC. The top phase (aqueous mixture and methanol) was transferred to a clean vial and maintained at – 40 ºC. The bottom organic phase was transferred to a clean vial and evaporated under a nitrogen gas flow. It was then preserved at – 40 ºC until injection. Internal standard contained a mixture of 13 deuterated lipidic standards at 100 µg mL^{-1} each: 15:0-18:1(d7) PC, 18:1(d7) Lyso PC, 15:0-18:1(d7) PE, 18:1(d7) Lyso PE, 15:0-18:1(d7) PG (Na Salt), 15:0-18:1(d7) PI (NH4 Salt), 15:0-18:1(d7) PS (Na Salt), 15:0-18:1(d7)-15:0 TAG, 15:0-18:1(d7) DAG, 18:1(d7) MAG, 18:1(d7) Chol Ester, d18:1-18:1(d9) SM, C15 Ceramide-d7.

Lipid extracts were resuspended in isopropanol:acetonitrile:ammonium acetate (10 mM) (50:30:20 vol) and diluted 50x in this same solution. Diluted extracts (500 µL) were then analyzed through direct infusion in an ESI qQTOF (TripleTOF 6600+, Sciex) mass spectrometer equipped with a DuoSprayTM source (Sciex). Samples were analyzed in both positive and negative ions mode with the MSMSALL acquisition mode, consisting on a TOF Ms scanning mode and a series of MSMS scanning modes stepped in mass intervals of 200-1200 umas. The conditions for the ionization source were optimized for both acquisition modes (nebulization gases GS1 10 psi and GS2 15 psi, curtain gas 20 psi, ion spray voltaje +5000/-4500 V, temperature 125 ºC and declustering potential of 80 V). MSMSALL acquisition mode was controlled by the Analyst software (Sciex). All precursors were selected in Q1, with an isolation window of 1Da in the MS interval of 200

to 1200 umas, where the precursor ions were equally distributed in the isolation windows. Colision energy for lipids in positive mode was CE 25 ± 15 V and CE -40 ± 15 V for those in negative mode. Data analysis was carried out by using the LipidView v1.3 software. Lipid classes and species were identified based on m/z exacta and fragmentation patterns.

10. Membrane permeability studies by flow cytometry

Flow cytometry studies were also carried out to examine the effect of SPIONs on cell membrane permeability. For this purpose, we employed two fluorescent dyes: FM^{TM} 1-43 and Laurdan. Conditions investigated included: (1) cells without SPIONs and without dyes (control), (2) cells exposed to NFA SPIONs without dye, (3) cells exposed to NFD SPIONs without dye, (4) cells loaded with FM1-43, (5) cells exposed to NFA SPIONs and loaded with FM1-43, (6) cells exposed to NFD SPIONs and loaded with FM1-43, (7) cells loaded with Laurdan probe, (8) cells exposed to NFA SPIONs and loaded with Laurdan and (9) cells exposed to NFD SPIONs and loaded with Laurdan. Briefly, right after isolation, ENPC suspensions were exposed to NFA and NFD SPIONs, as appropriate, at a concentration of 0.05 mg mL^{-1} for 2 h in a sterile incubator at 37 ºC under a CO_2 atmosphere (5%). After treatment, cells were centrifuged at 300 *g* for 4 min, the supernatants removed, and the pellets suspended in 1 mL of either Hank's without calcium and magnesium (FM1-43) or Dulbecco's Modified Eagle Medium (DMEM; Laurdan). For FM1-43 staining, cells were filtered using 12 x 75 mm tubes with a 35 µm nylon cell strainer Cap (Falcon) to remove cell aggregates and centrifuged at 300 *g* for 4 min at 4 ºC. Next, supernatants were discarded, and pellets were suspended in 1 mL of Hank's without sodium and magnesium. Subsequently, 2.5 µl of FM1-43 (2.5 µg mL^{-1}) were added to the samples and incubated for 1 min. Labelled samples were then analyzed by using flow

cytometry. FM1-43 fluorescence was excited with a 488 nm laser and the emission fluorescence was collected with a V585/42 detector in the 564-606 nm. For Laurdan staining, cell pellets were incubated with 5 µl of Laurdan (5 µM) for 1 h at 37 ºC. Then, cells were filtered using the same tubes as before and centrifuged at 300 *g* for 4 min at 4 ºC. Supernatants were discarded, pellets suspended in 1 ml of DMEM and measured by flow cytometry immediately after. Laurdan fluorescence was excited with a 405 nm laser and the fluorescence emission was detected with two different detectors: V450/50 for collecting emission at 425-475 nm and V530/30 for collecting emission at 515-545 nm. The ratio between the emissions at 530/30 and 450/50 was calculated. General Polarization (GP) values were calculated using the median of fluorescence intensity (MFI) emission values at 450 nm and 530 nm and excitation at 405 nm, according to the formula $^{405}GP_{ex} = (MFI_{450} - MFI_{530})/(MFI_{450} + MFI_{530})$. All samples were analyzed by using a FACS Canto II cytometer (BD Biosciences) within 30 min after staining and recorded for 2 min with at least 10,000 events recorded in the FSC gate. Flow cytometry data analysis was carried out by using the FlowJo 10.7 software (BD Biosciences).

The gating strategy followed for FM1-43 studies is illustrated in **Figure S2**. In detail, after cell aggregates exclusion, singlets gate was plotted on an FSC *vs* SSC dot-plot and FSC gate was created to exclude cellular debris. Next, FSC-gated events, plotted on a FSC *vs* SSC dot-plot, were subdivided in three different regions: FSCdim (*i.e.* diminished FSC and SSC signals), A subset (*i.e.* events that present high values of SSC) and B subset (*i.e.* events with lower or intermediate values of SSC). Subsequently, FM1-43 fluorescence was measured for all three subpopulations and two different regions were defined: a FM1-

43$^+$ region (*i.e.* higher levels of FM1-43 fluorescence) and a FM1-43^{low} region (*i.e.* events with a lower fluorescence for FM1-43).

The gating strategy followed for Laurdan studies is illustrated in **Figure S3**. In detail, after cell aggregates exclusion, singlet events were plotted on an FSC *vs* SSC dot-plot and FSC gate was created to exclude cellular debris. As samples treated with SPIONs displayed an increased number of events without fluorescence or with a low Laurdan fluorescence, and a diminished FSC signal, events with Laurdan fluorescence corresponding to membrane stained events were gated in a Laurdan V530/30 *vs* FSC dot-plot as cells. Next, cell-gated events plotted again on a FSC *vs* SSC dot-plot were subdivided in A and B subsets as in FM1-43 studies based in SSC values. Median fluorescence intensities (MFI) collected at V530/30 nm (liquid phase) and V450/50 nm (gel phase) were compared in A and B subsets for all conditions tested.

11. RT-qPCR studies

RNA concentration and purity were quantified in a Nanodrop One spectrophotometer (Thermo Fisher Scientific) and a Quantus fluorometer (Promega Corporation). Yield range was between 40.35 - 75.35 ng μL^{-1} and 39.20 - 82.20 ng μL^{-1}, respectively. Retrotranscription (RT) reactions were performed using the iScript cDNA Synthesis kit (Biorad PN170-8891) following manufacturer´s instructions. Briefly, 500 ng of total RNA from each sample were combined with 5 µL of master mix (with all necessary reagents including a mixture of random primers and oligo-dT for priming). The reaction volume was completed up to 20 µL with DNAse/RNAse free distilled water (Gibco PN 10977). Thermal conditions consisted of the following steps: 5´ x 25 °C, 20´ x 46 °C and 1´

x 95 °C. The genes of interest (GOIs) analyzed were *Fth1*, *Ireb2*, *Slc11a2*, and *Tfrc*. The putative reference gene analyzed was 18S. The ValidPrime Universal kit (Fw: GATTCAAAAAGTCCAGTCCC; Rv: TAGTATCTCCCCACCAAAA) was used as control for genomic background. Primer sequences used are included in supporting information as **Table S1**. qPCR reactions were performed in 384-well plates in a 10 µL final volume (4 µL of sample + 6 µL of mastermix and primers), as follows:

- Samples: Non-template control (NTC; 0 µL sample + 4 µL H_2O) and unknown samples (0.2 µL (5 ng cDNA) + 3.8 µL H_2O).

- Primers and mastermix: 1 µL of primer mix (5 µM of each primer) + 5 uL Sso Fast EvaGreen Supermix (Biorad, CN 172-5204) which includes Sso7d-fusion® DNA Polymerase, dNTPs and the rest of reagents needed to perform the qPCR.

RTs were set up manually. The rest of reactions were set up robotically, with an Eppendorf pipetting robot (epMotion 5075). qPCR reactions were performed in a CFX384 Real Time System C1000 Thermal Cycler (Bio-Rad), in hard-Shell® 384-Well PCR Plates White Well Clear shell (Bio-Rad CN HSP-3805). Thermal conditions depended on used mastermix and consisted of Sso Fast EvaGreen Supermix: 30´´ x 95 °C + (5´´ x 95 °C and 5´´ x 60 °C) x 40. qPCR reactions were performed in a CFX384 Real Time System C1000 Thermal Cycler (Bio-Rad). We also included a Melting curve from 60 °C to 95 °C (0.5 °C s^{-1}) at the end of the program to verify the specificity of the PCR. Fluorescence was acquired during both the 60 °C and Melting steps. The assay specificity for all tested genes was confirmed by their unique Melting peaks with indicated mastermix for each amplicon. In order to discard a potential contamination of reagents and/or primer-dimer artifacts, a

non-template control (NTC) was carried out using all the reagents except the sample. For all tested genes, NTC amplifications were always negative or delayed more than 5 cycles with respect to the experimental samples, which allowed ruling out contamination and primer-dimer artifacts. Technical triplicates were performed in order to correct pipetting errors in plate loading. In general, accurate technical repeats were obtained. In order to test for the presence of gDNA in RNA samples, we performed the Valid Prime assay, which measures the gDNA contribution to the qPCR signal using an optimized gDNA-specific assay (VPA) and a gDNA reference sample in this case. The VPA, targeting a non-transcribed locus that is present in exactly one copy per haploid normal genome, is used to measure gDNA contents in RT samples and the gDNA reference is used to normalize for GOI-specific differences in gDNA sensitivity. These measures provide a reliable correction for gDNA background in qPCR. Correction is possible for any GOI assay that consistently amplifies gDNA, given that the DNA contribution does not exceed 60 % of the signal. In this work, no correction is necessary because VAP gene has no signal except for gDNA sample. Data processing was carried out using the software GenEx v. 5.4.4 (MultiD Analyses AB, Gothenburg, Sweden), performing the subsequent steps: (1) Efficiency correction; (2) Average technical qPCR replicates, (3) Normalization with selected reference gene, and (4) Relative quantification $2^{-\Delta(\Delta Cq)}$,[45] being ΔCq = Cq value of each individual sample against the Cq value of calibrator group (first biological replicate of the NFA SPION group). An appropriate normalization strategy is essential to correct the experimental variability (*e.g.* integrity differences, pipetting errors). In this study, the putative reference gene analyzed was 18S.

12. Statistics

Results were expressed in conventional bar graphs as the mean ± standard error of the mean (SEM), unless otherwise indicated, of at least three independent experiments for each assay ($N \geq 3$). Statistical analysis was performed by using the IBM SPSS Statistics software (version 28.0.1.0). Comparisons among groups were done by one-way analysis of variance (ANOVA) followed by either post-hoc Scheffé, Tukey HSD or Games-Howell tests (homogeneous vs. heterogeneous variances as dictated by Levene's test). Comparisons between two groups, when needed, were carried out by *T* test. In all cases, the significance level was defined as $p < 0.05$.

Supplementary Figures

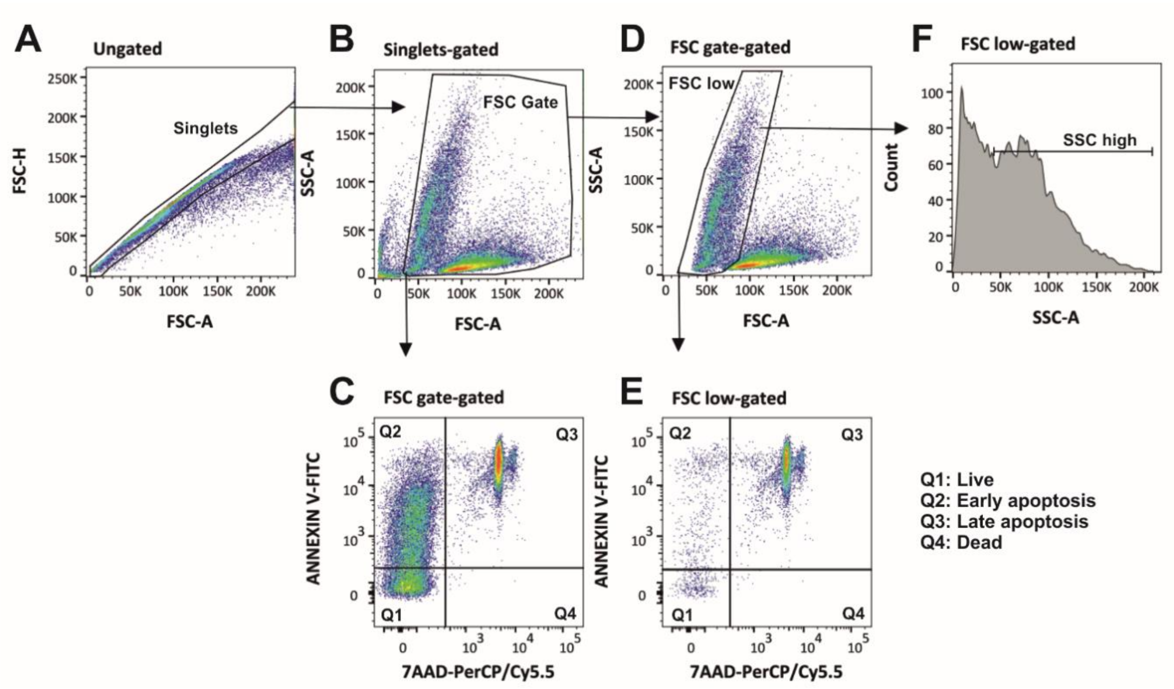

Figure S1. Scheme illustrating the gating and analysis strategies followed in flow cytometry studies.

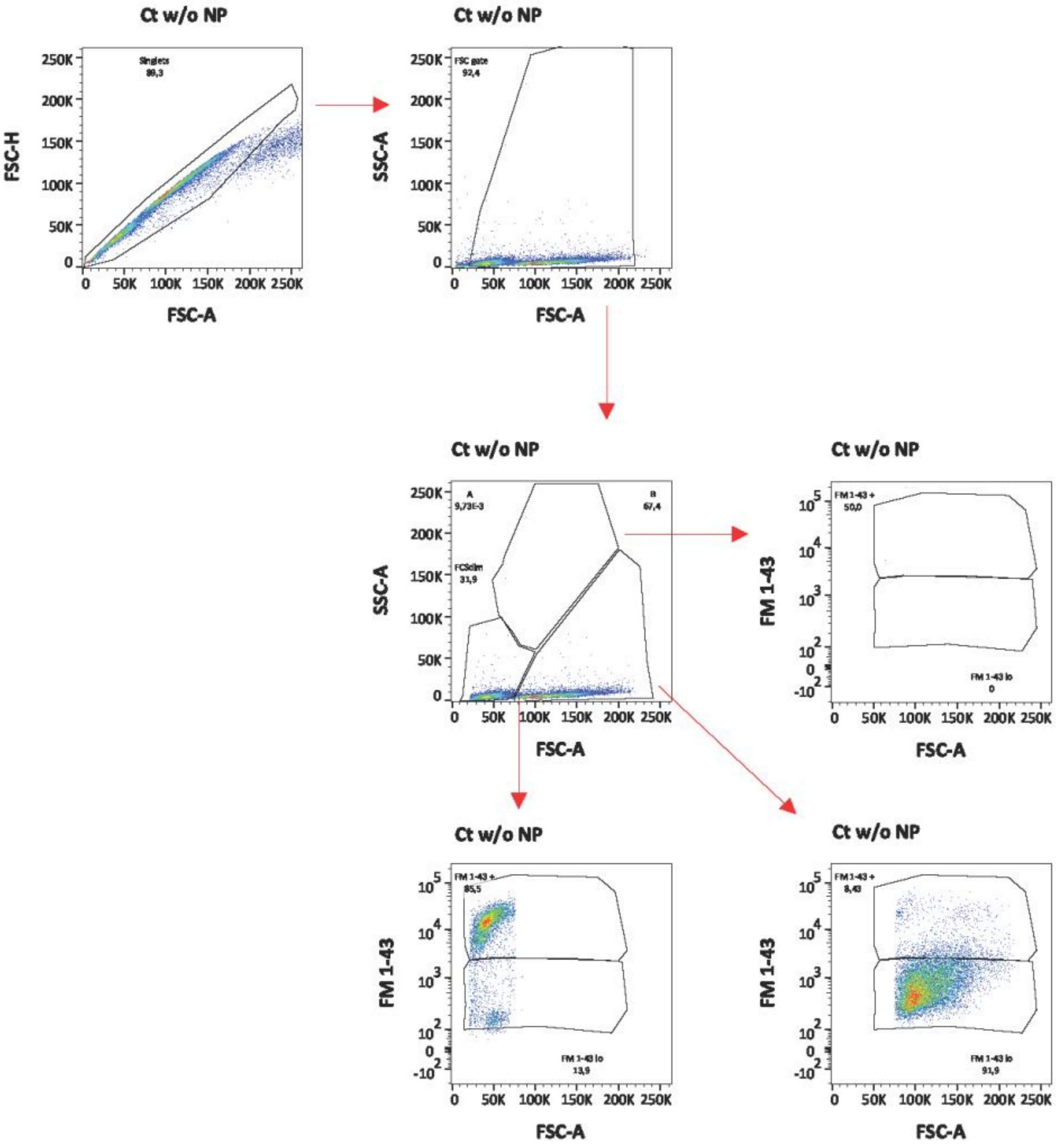

Figure S2. Scheme illustrating the gating and analysis strategies followed in flow cytometry studies with the FM^{TM} 1-43 probe.

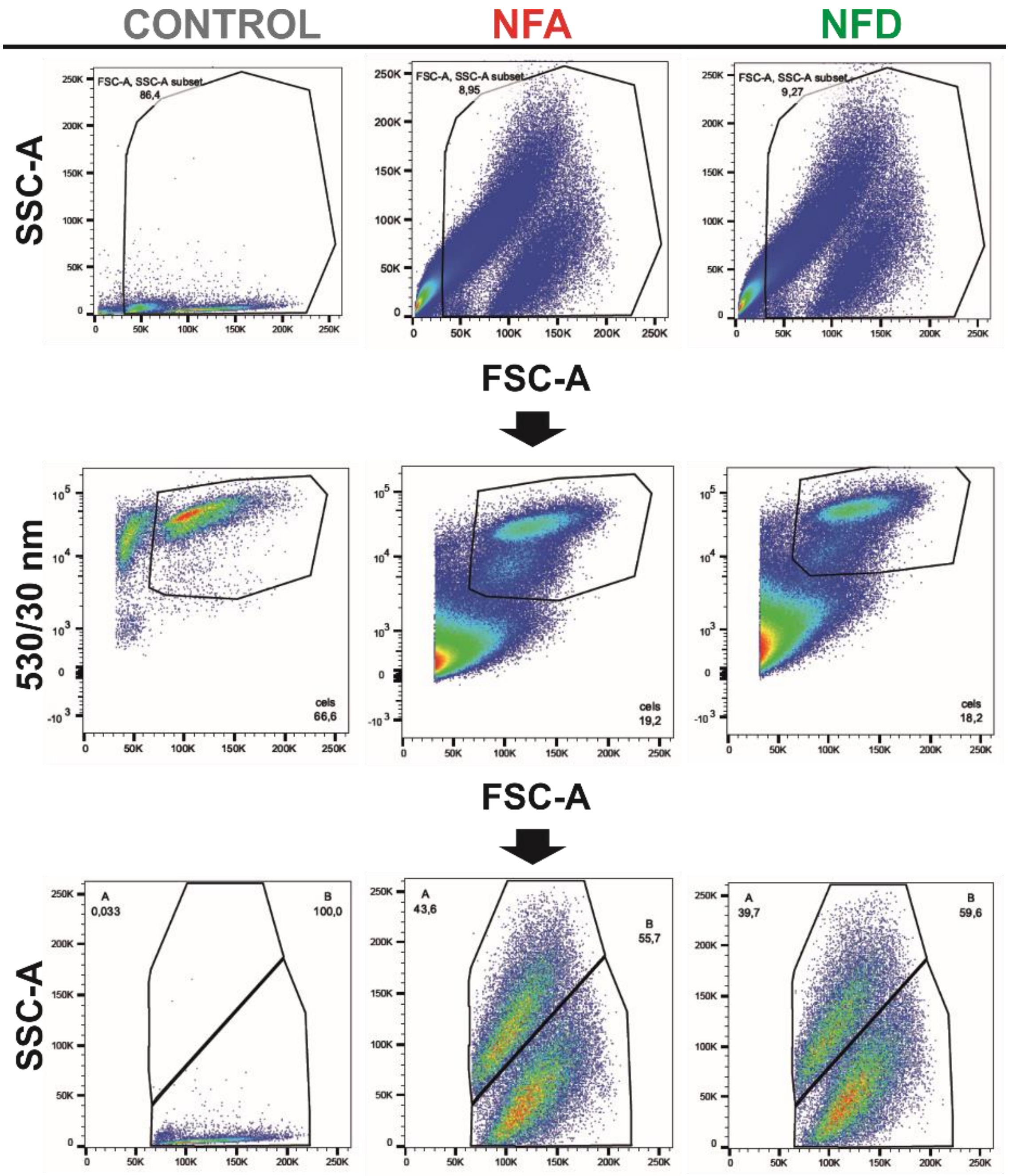

Figure S3. Flow cytometry strategy followed for the selection of subsets A and B in ENPC suspensions incubated with SPIONs in studies with the Laurdan dye.

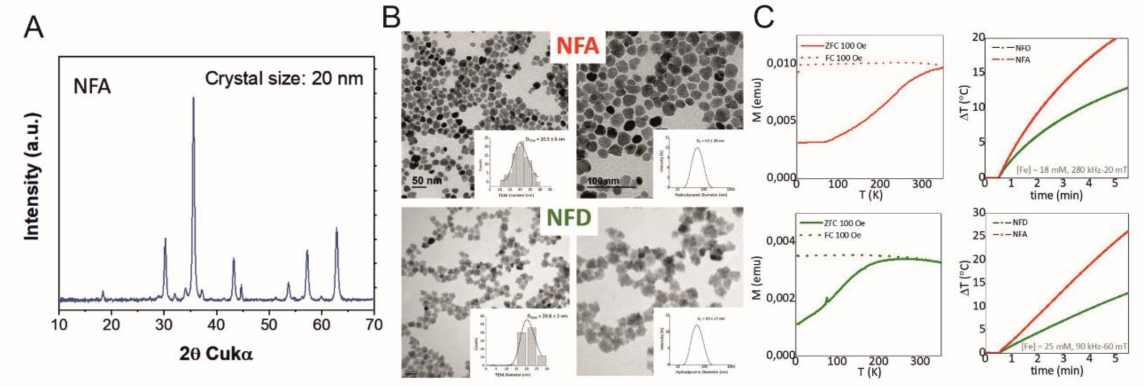

Figure S4. Physico-chemical properties of SPIONs obtained in autoclave (NFA) or in an open system (NFD). XRD data (A), structural (TEM images at different magnifications), colloidal (DLS data in intensity) (B), and magnetic properties (Zero Field Cooling -ZFC- and Field Cooling -FC- magnetization curves (left) and temperature increase with time (right) under an AMF at two different field conditions) (C). Graph insets in (B) show TEM size distribution (left) and hydrodynamic size distribution calculated by DLS (right).

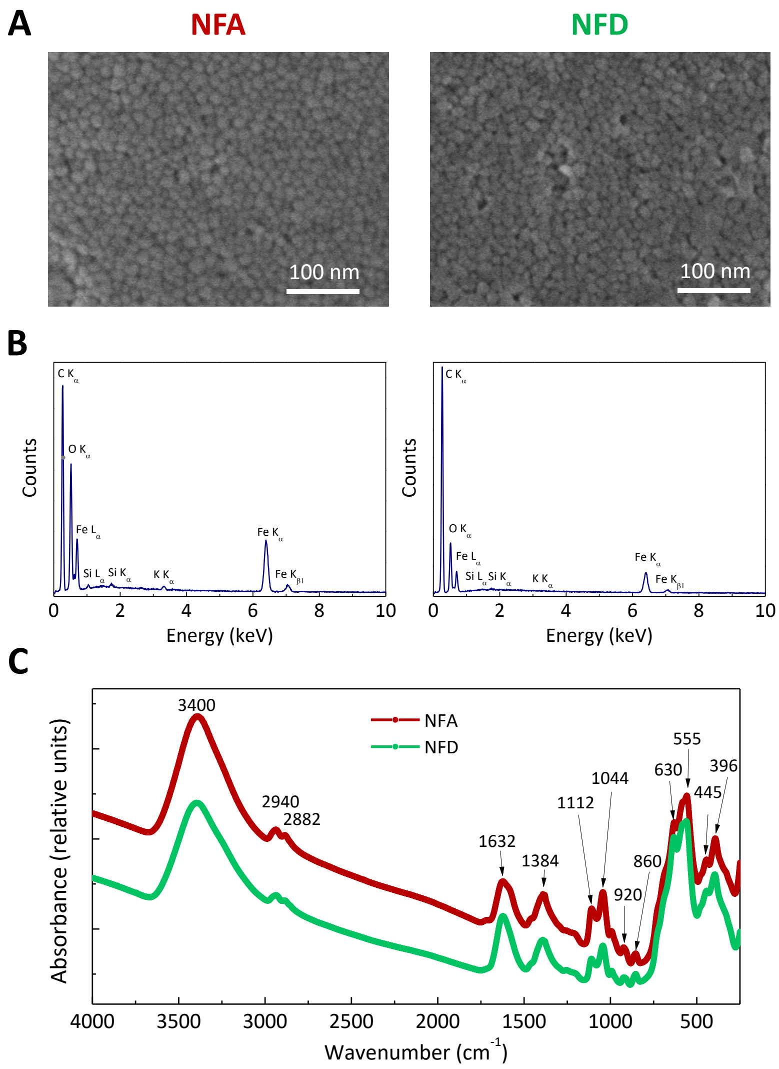

Figure S5. (A) Representative scanning electron microscopy (SEM) images of NFA and NFD SPIONs. (B) Energy-dispersive X-ray (EDX) profiles confirming the elemental composition of both SPIONs, discarding the presence of eventual contaminants. Analyses were conducted at 2 kV. (C) Infrared spectra for NFA (red) and NFD (green) samples: O-H stretching vibrations due to water (3400 cm^{-1}), C-H doublet symmetric and asymmetric stretching (2940 and 2882 cm^{-1}) and carboxylic groups (1632 and 1384 cm^{-1}) due to the citric acid coating and, finally, C-O-C vibrations (1112 and 1044 cm^{-1}) and primary alcohols (860 and 920 cm^{-1}) assigned to some polyol rests from the synthesis.

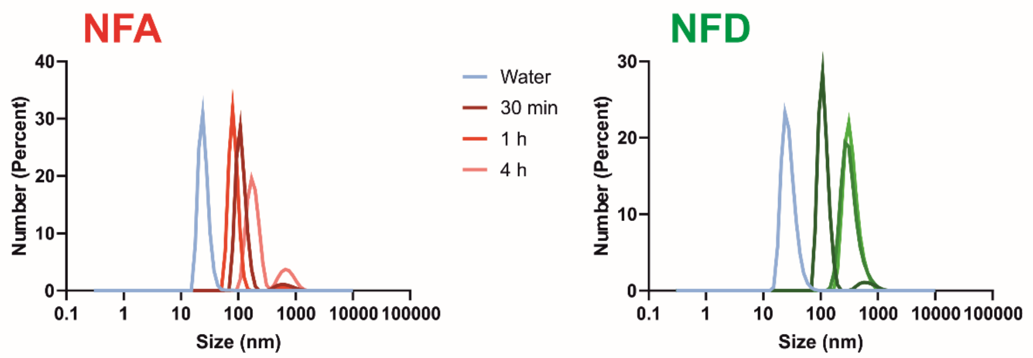

Size (nm)	NFA_NB⁺ 30 min	NFA_NB⁺ 1 h	NFA_NB⁺ 24 h
Number	108.0	79.9	169.9
Intensity	125.6	92.9	197.6

Size (nm)	NFD_NB⁺ 30 min	NFD_NB⁺ 1 h	NFD_NB⁺ 24 h
Number	68.7	267.2	310.7
Intensity	79.9	488.7	488.7

Figure S6. Dynamic light scattering (DLS) measurements of the hydrodynamic size (in number) of NFA (red) and NFD (green) SPIONs when dispersed in distilled water and incubated in complete Neurobasal culture media for 30 min, 1 h and 4 h at 37 ºC in a sterile incubator. Size expressed in number and intensity are summarized in the tables under respective graphs.

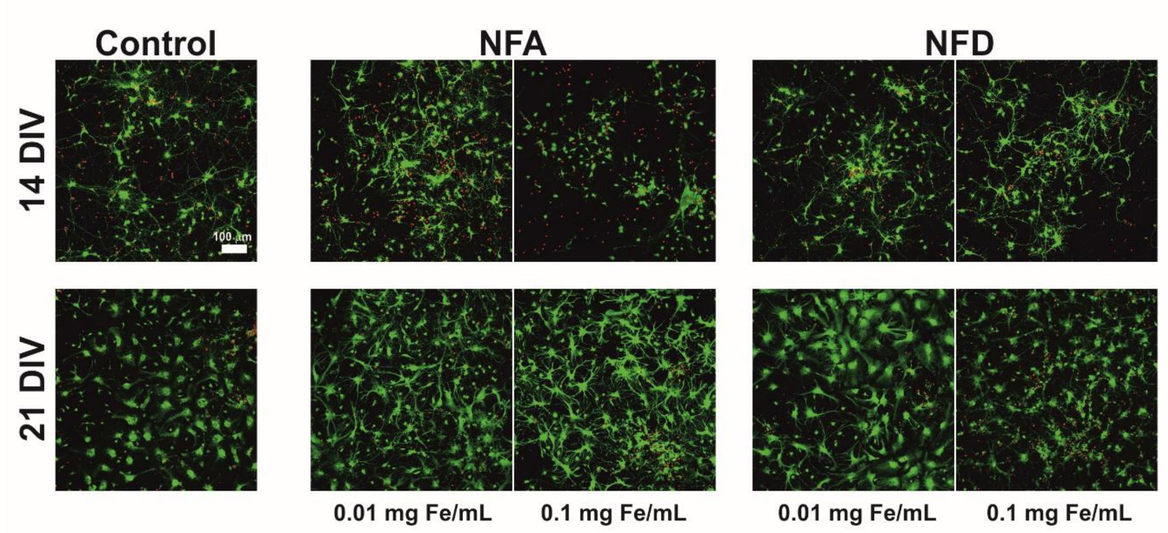

Figure S7. Representative CLSM images illustrating live (green) and dead (red) cells in ENPC cultures exposed to NFA and NFD SPIONs at 14 and 21 DIV. Scale bar: 100 µm.

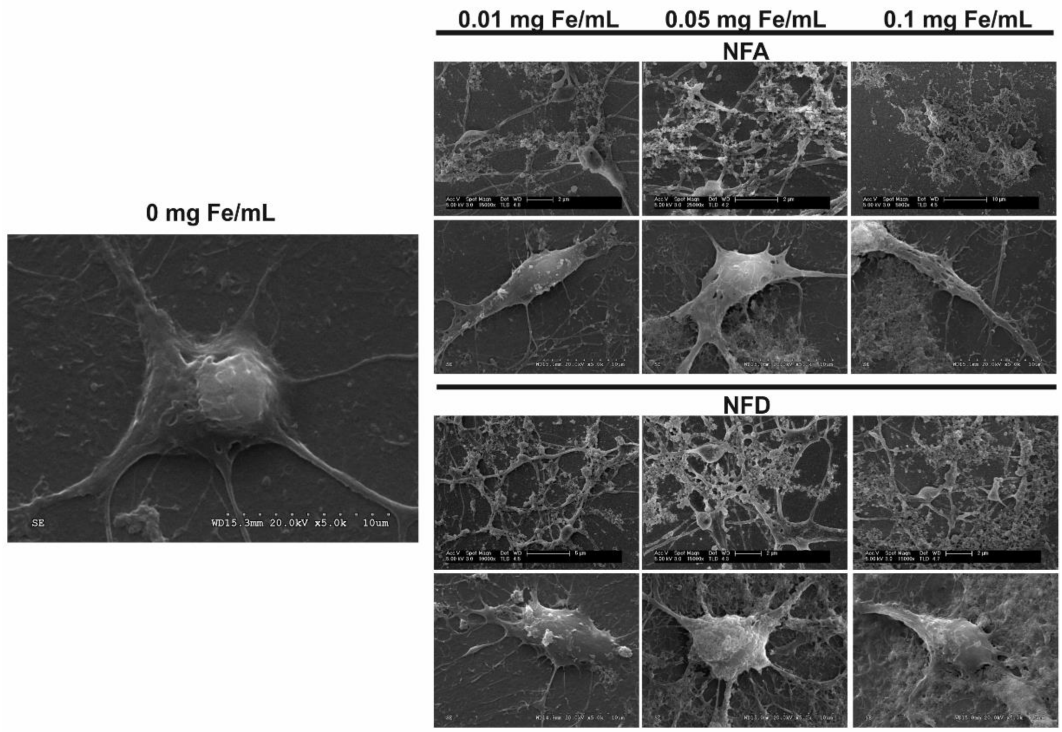

Figure S8. Representative SEM images of detailed surface interaction of ENPCs with SPIONs at different concentrations.

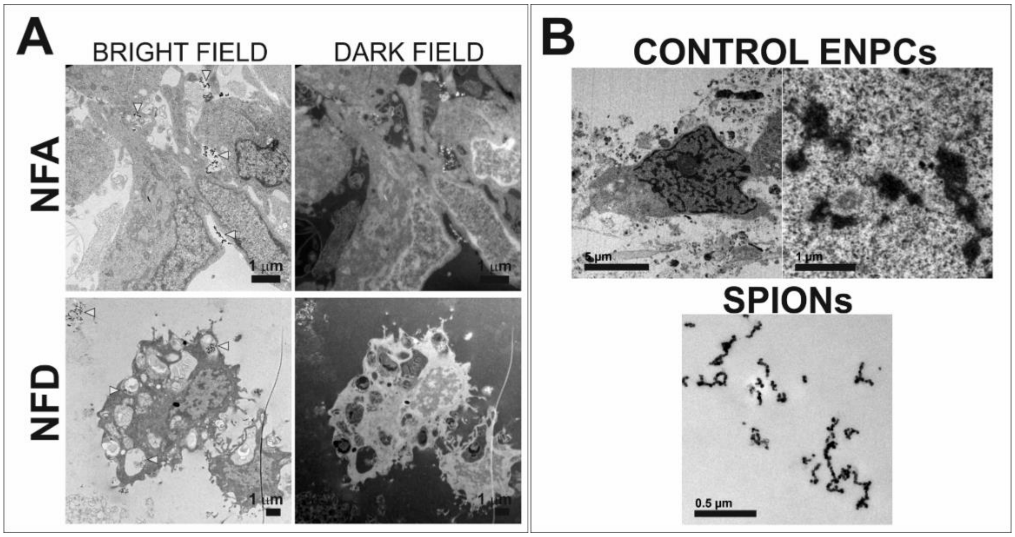

Figure S9. (A) TEM images acquired under bright (left) and dark (right) field conditions to illustrate the clearly identifiable nature of SPIONs as brighter elements in comparison to intracellular components and eventual contaminants from sample preparation. **(B)** Representative TEM images of NFA SPIONs and control ENPCs for comparison. Scale bars are indicated in each image.

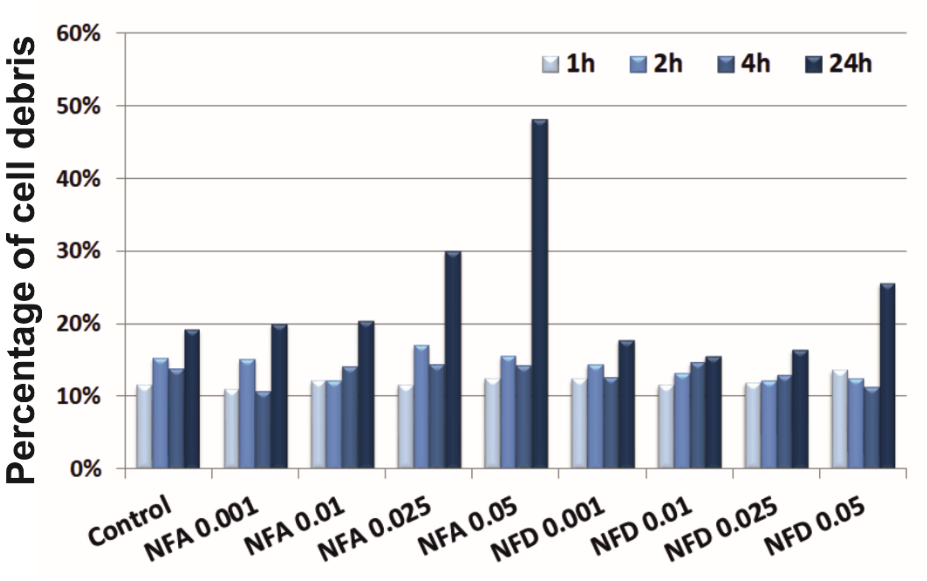

Figure S10. Percentage of cell debris for ENPC suspensions exposed to SPIONs under the different conditions tested.

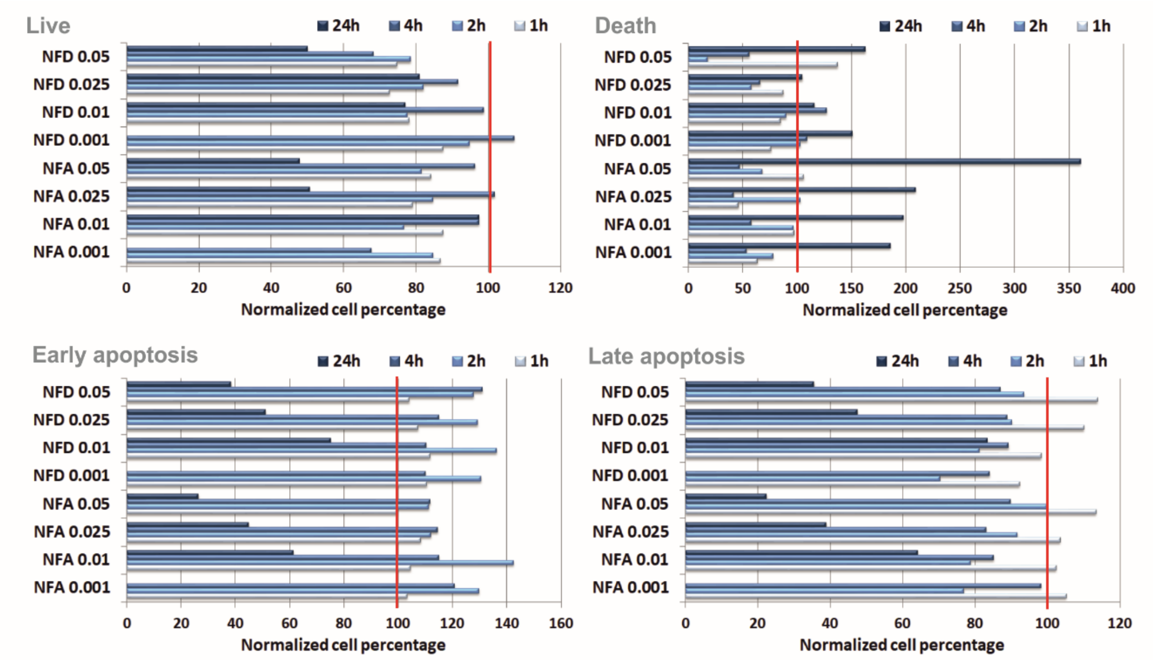

Figure S11. Characterization of the combined effect of SPION type, concentration and exposure time on primary neural cell complexity by flow cytometry. Normalized cell percentages for live, early apoptosis, late apoptosis and dead cells are shown for the different conditions tested. The red line indicates the value for control cells without SPIONs.

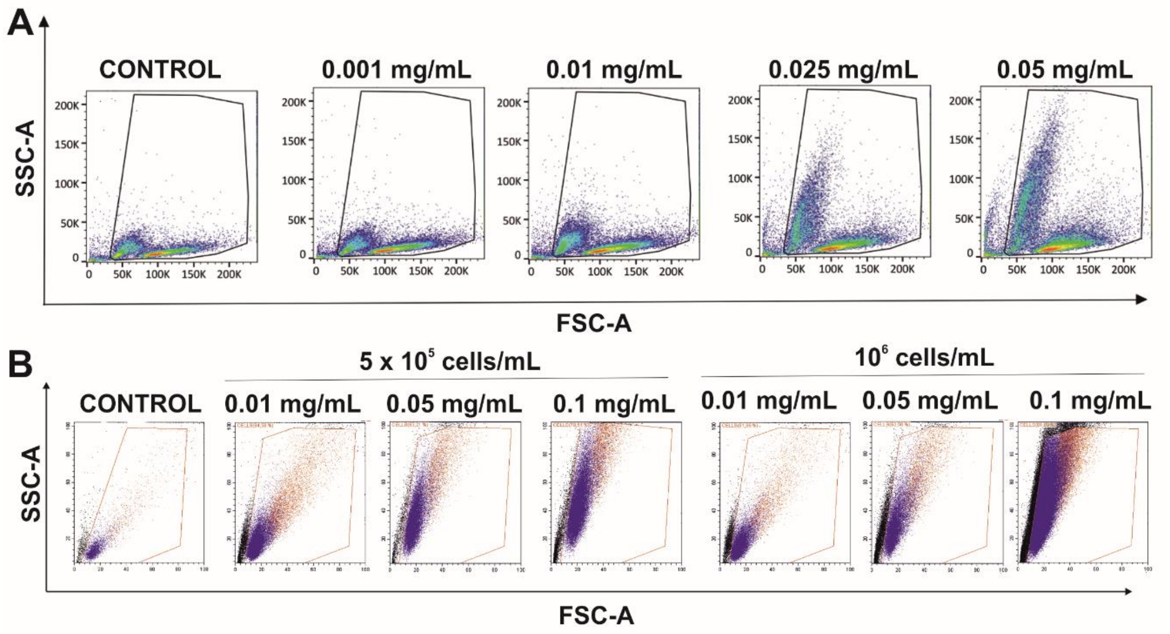

Figure S12. SSC/FSC dot plots illustrating evident changes in cell complexity and size, respectively, for suspensions of ENPCs (A) and L929 fibroblasts (B) exposed to different concentrations of NFA SPIONs.

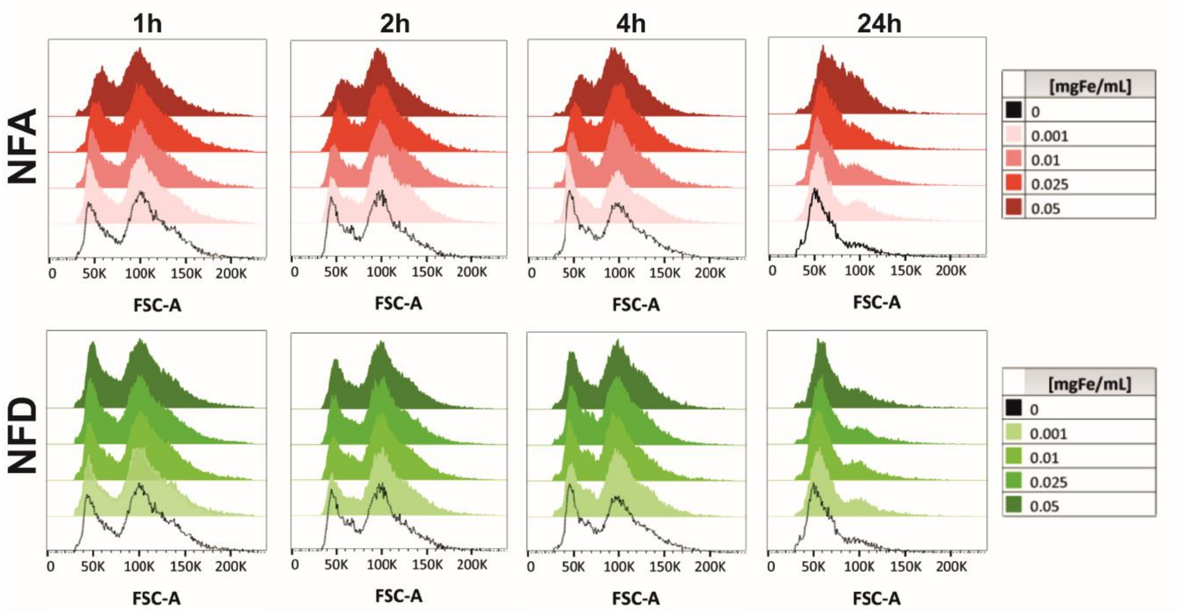

Figure S13. Histogram overlays of FSC values for ENPC cultures exposed to different types of multicore SPIONs at different concentrations and exposure times.

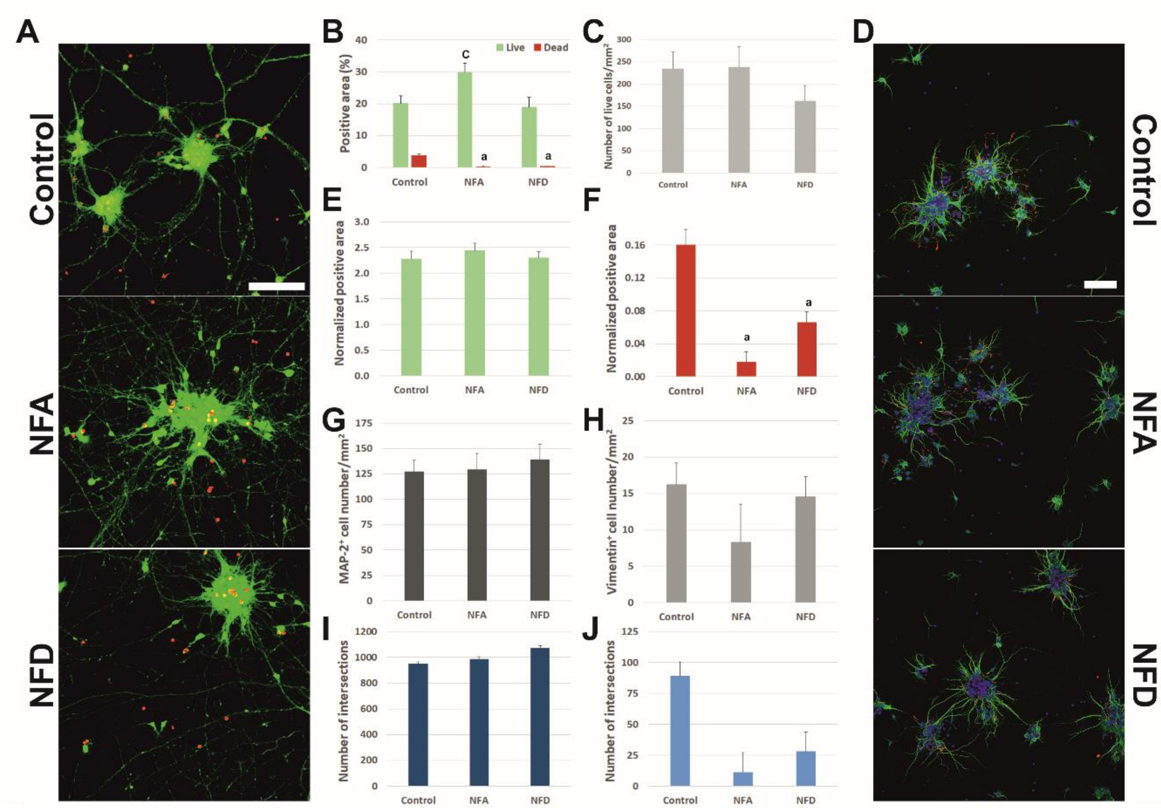

Figure S14. Evaluation of cell viability and neural differentiation on SPION-treated ENPC suspensions cultured for 7 DIV. **(A)** Representative CLSM images illustrating live (green) and dead (red) cells. Scale bar: 100 µm. **(B)** Histogram for the positive area covered by live and dead cells. **(C)** Number of live cells per area unit. **(D)** Representative CLSM images illustrating neurons (green, MAP-2$^+$) and non-neuronal cells including glial cells (red, vimentin$^+$) cells. Cell nuclei are stained with Hoechst (blue). Scale bar: 100 µm. Normalized positive area for MAP-2$^+$ cells **(E)** and vimentin$^+$ cells **(F)**. Number of MAP-2$^+$ cells **(G)** and vimentin$^+$ cells **(H)**. Number of intersections for MAP-2$^+$ cells **(I)** and vimentin$^+$ cells **(J)**. Statistics: *$p < 0.05$, with respect to control samples (*a*) and NFD (*c*).

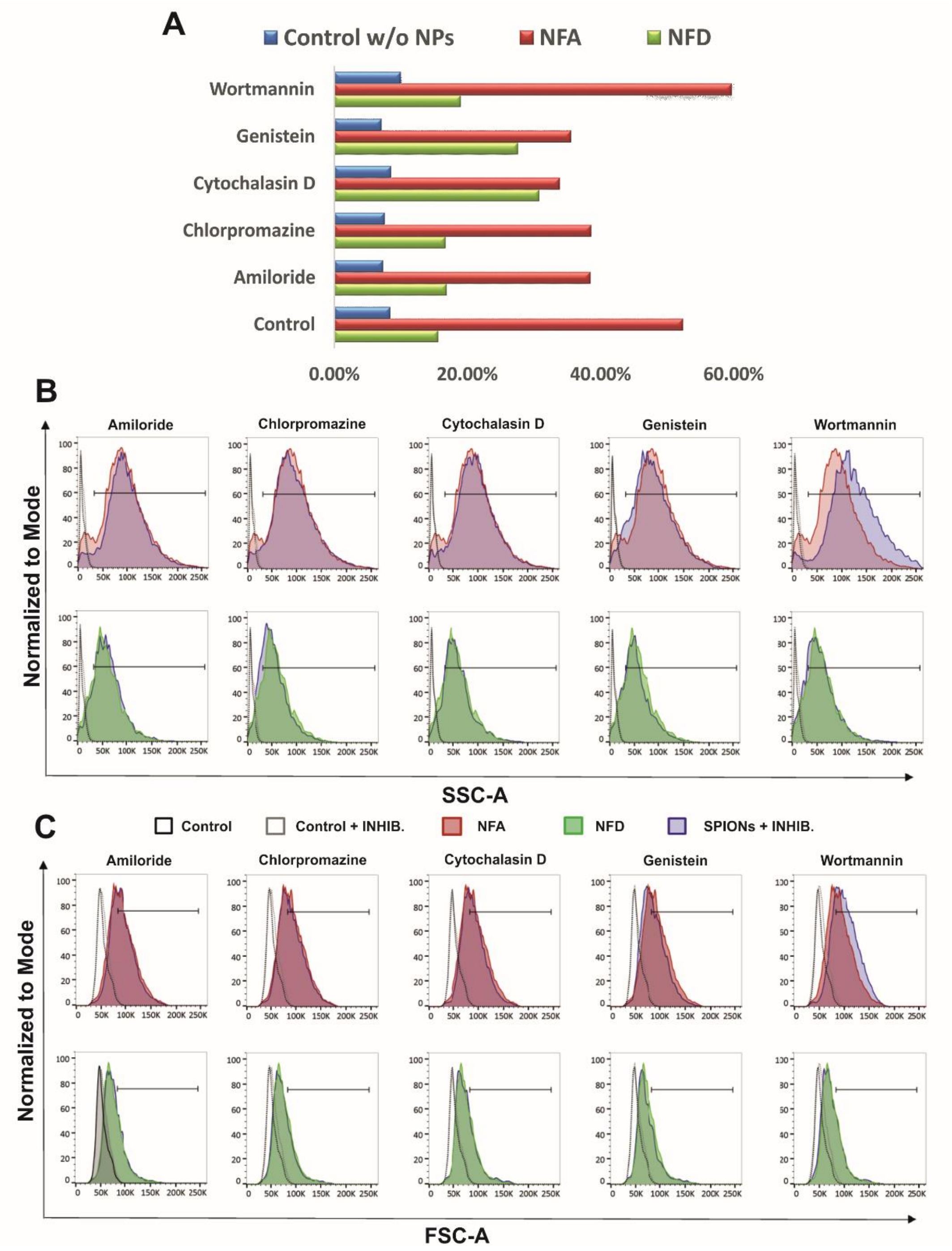

Figure S15. Elucidation of the internalization route for NFA and NFD SPIONs in primary neural cells by the use of molecular inhibitors and flow cytometry. **(A)** Percentages of cell debris. **(B)** SSC and **(C)** FSC histogram overlays for the SSC^{high} cell population in ENPC suspensions exposed to different internalization inhibitors. Cells were exposed to SPIONs at 0.05 mg Fe mL^{-1} for 2h.

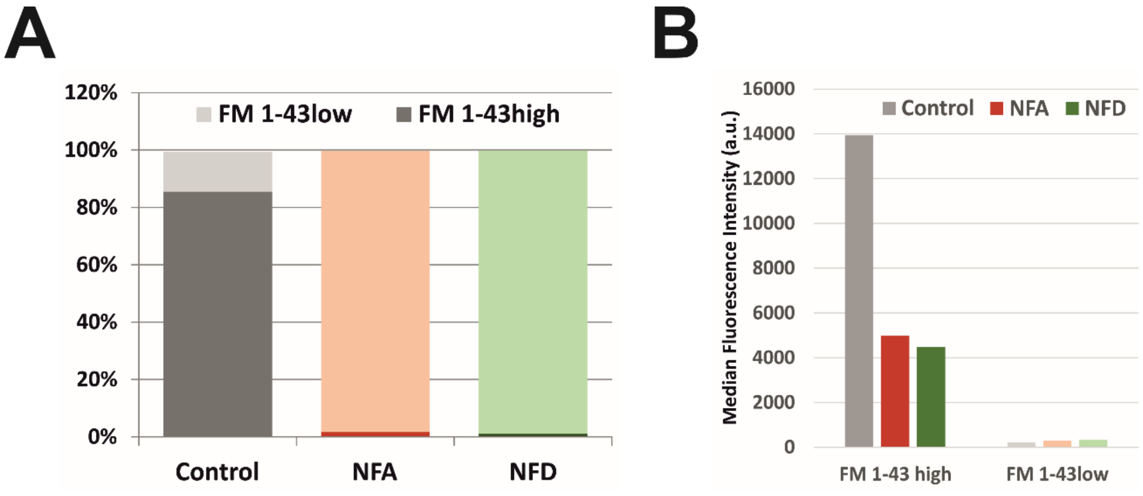

Figure S16. Percentage (A) and median of fluorescence intensity (B) of events gated as FSC^{dim} in flow cytometry studies with ENPCs incubated with FM1-43.

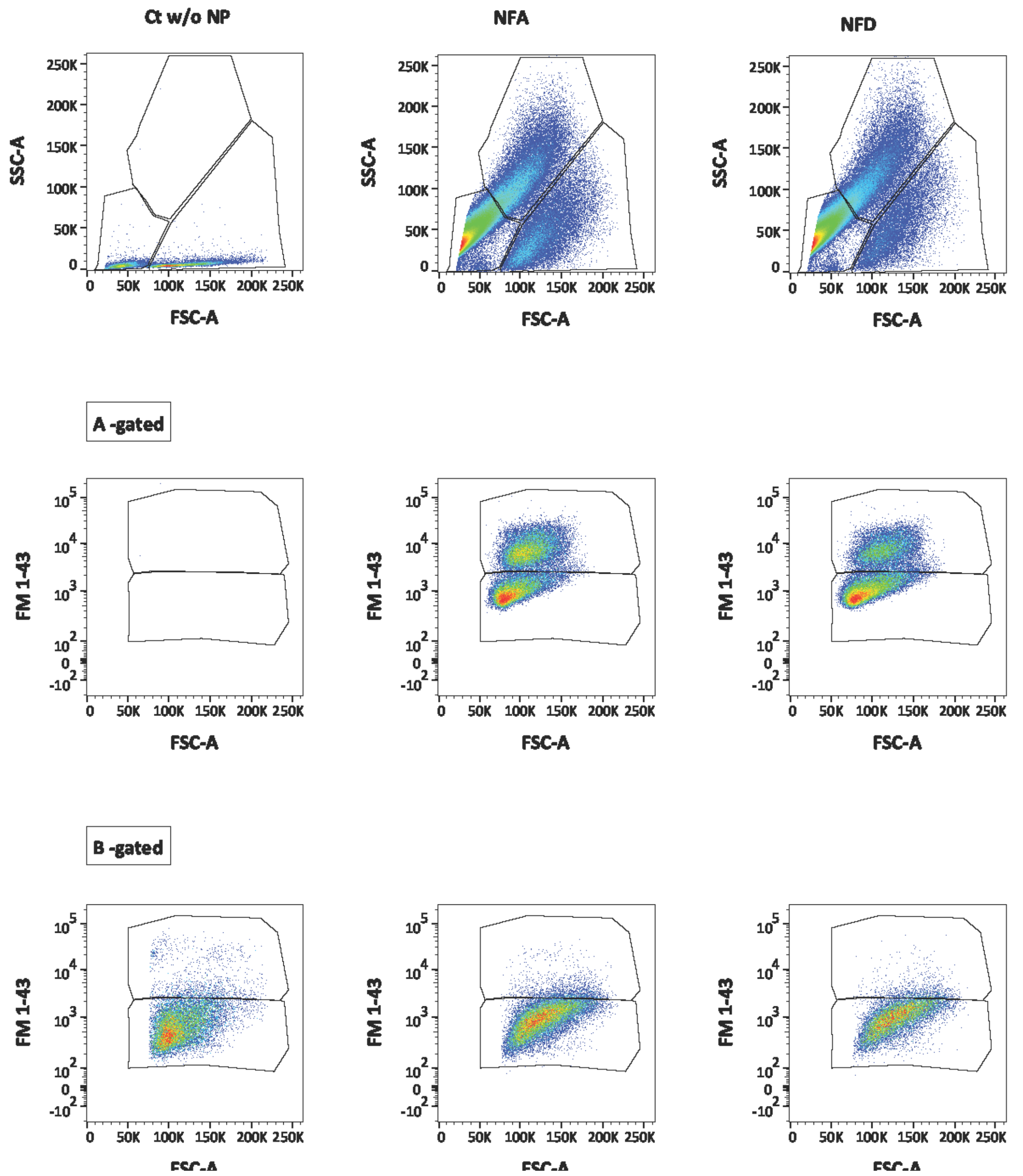

Figure S17. Representative flow cytometry dot plots from ENPCs incubated with FM1-43. Gating selection from SSC/FSC dot plots (top) and respective FM1-43/FSC dot plots for subsets A (middle) and B (bottom). Treatment groups are illustrated in ordered columns from left to right as follows: control, NFA- and NFD-treated cells.

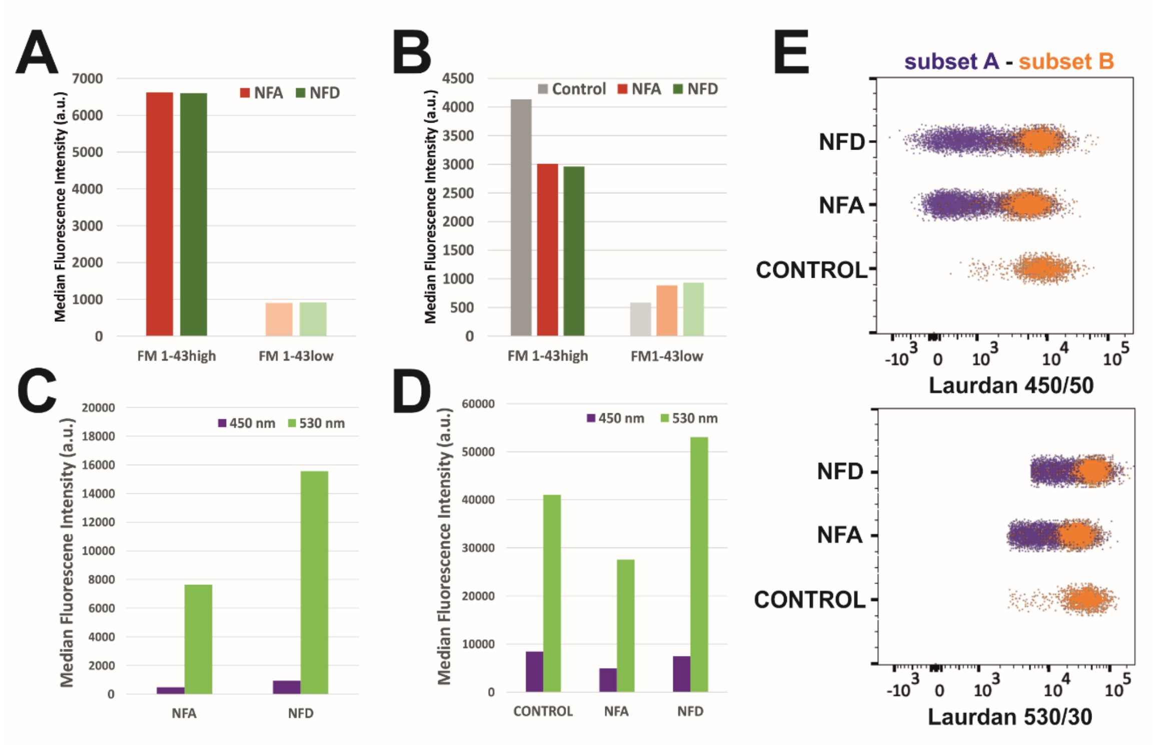

Figure S18. Median fluorescence intensity (MFI) values for cells incubated with the FM 1-43 probe for subsets A **(A)** and B **(B)** and cells incubated with the Laurdan probe for subsets A **(C)** and B **(D)** at both fluorescence ranges (450/50 for the gel state and 530/30 for the liquid state). **(E)** Dot plots for both populations after incubation with Laurdan.

Table S1. Primer sequences used in RT-qPCR studies.

Gene	Primer	Sequence 5'-3'	Amplicon Size	Design
Fth1	Fw	CAGCGAGGTGGACGAATCTT	83 pb	IS
	Rv	CACTCCATTGCATTCAGCCC		
Slc11a2	Fw	GGCATGTGGCACTGTATGTG	150 pb	IS
	Rv	CGCTCAGCAGGACTTTAGAGAT		
Ireb2	Fw	GGCGATGGACTCCCCAAGTG	141 pb	IS
	Rv	AGGACCCGTATTGAGTAAGGCA		
Tfrc	Fw	CCGGCCTATATGCTTGGGTAG	138 pb	IS
	Rv	TGCTGATCTGGCTTGATCCAT		

Table S2. Main lipid classes for the different treatment groups as measured by shotgun lipidomics. Concentration values are indicated as signal intensity (cps). Mean ± SEM of three independent experiments.

Category	Class	CONTROL	AMF	NFA	NFD	NFA +AMF	NFD +AMF
GPL	Phosphatidylcholine (PC)	174915 ± 15380	229161 ± 856	225133 ± 10058	209815 ± 968	270443 ± 17146	257816 ± 7781
		**ANOVA p < 0.001***; control *vs* NFA+AMF (p = 0.002**), control *vs* NFD+AMF (p = 0.005**), NFD *vs* NFA+AMF (p = 0.047*)**					
GPL	Phosphatidylethanolamine (PE)	17228 ± 1307	19923 ± 953	20991 ± 2215	20998 ± 303	22824 ± 927	31219 ± 2906
		**ANOVA p = 0.001**; control *vs* NFD+AMF (p = 0.003**), AMF *vs* NFD+AMF (p = 0.015*), NFA *vs* NFD+AMF (p = 0.029*), NFD *vs* NFD+AMF (p = 0.029*)**					
GPL	Phosphatidylserine (PS)	7734 ± 650	8484 ± 2	7095 ± 171	6629 ± 270	7768 ± 28	8563 ± 178
		**ANOVA p = 0.005**; AMF *vs* NFD (p = 0.029*), NFD vs NFD+AMF (p = 0.022*)**					
GPL	Phosphatic acid (PA)	3889 ± 1003	2740 ± 57	2324 ± 116	1810 ± 328	2087 ± 334	2486 ± 579
		**ANOVA p = 0.149**					
GPL	Phosphatidylinositol (PI)	4036 ± 502	3245 ± 423	2648 ± 223	3621 ± 11	2948 ± 40	3369 ± 710
		**ANOVA p = 0.273**					
FA	Free fatty acids	17078 ± 423	12120 ± 356	8424 ± 168	8299 ± 5	6749 ± 358	5633 ± 362
		**ANOVA p < 0.001***; control *vs* all groups (p < 0.001***), AMF *vs* all groups (p < 0.001***), NFA *vs* NFD+AMF (p = 0.002**), NFD *vs* NFD+AMF (p = 0.003**)**					
SL	Sphingomyelin (SM)	136582 ± 6232	156580 ± 1331	151609 ± 1826	141391 ± 1324	174979 ± 4057	184544 ± 2632
		**ANOVA p < 0.001***; control *vs* AMF (p = 0.036*), control *vs* NFA+AMF (p < 0.001***), control *vs* NFD+AMF (p < 0.001***), AMF *vs* NFD+AMF (p = 0.003**), NFA *vs* NFA+AMF (p = 0.013*), NFA *vs* NFD+AMF (p < 0.001***), NFD *vs* NFA+AMF (p < 0.001***), NFD vs NFD+AMF (p < 0.001***)**					
SL	Ceramide (Cer)	906 ± 312	950 ± 229	874 ± 131	413 ± 88	447 ± 64	328 ± 28
		**ANOVA p = 0.073**					
GL	Triacylglyceride (TAG)	1450 ± 41	261 ± 33	511 ± 23	852 ± 259	383 ± 9	254 ± 27
		**ANOVA p < 0.001***; control *vs* NFD (p = 0.002**), control *vs* NFD+AMF (p < 0.001***), AMF *vs* NFD (p = 0.005**), AMF *vs* NFD+AMF (p < 0.001***), NFA *vs* NFD (p = 0.001**), NFA *vs* NFD+AMF (p < 0.001***), NFD *vs* NFD+AMF (p < 0.001***), NFA+AMF *vs* NFD+AMF (p < 0.001***)**					
StL	Cholesterol ester	171 ± 9	160 ± 8	175 ± 10	91 ± 6	127 ± 6	462 ± 12
		**ANOVA p < 0.001***; control *vs* AMF (p < 0.001***), control *vs* NFD (p = 0.002**), control *vs* NFA+AMF (p < 0.001***), control *vs* NFD+AMF (p < 0.001***)**					

FA: fatty acyls; GL: glycerolipids; GPL: glycerophospholipids; SL: sphingolipids; StL: sterol lipids.
